# Single-molecule imaging of small aggregates of IAPP in type 2 diabetes serum with rationally-designed antibody-like scaffolds

**DOI:** 10.1039/d5sc01427a

**Published:** 2025-12-11

**Authors:** Jiacheng Lin, Yu P. Zhang, Sean Chia, David Klenerman, Pietro Sormanni, Michele Vendruscolo

**Affiliations:** a Centre for Misfolding Diseases, University of Cambridge Cambridge CB2 1EW UK ps589@cam.ac.uk mv245@cam.ac.uk; b Yusuf Hamied Department of Chemistry, University of Cambridge Cambridge CB2 1EW UK dk10012@cam.ac.uk

## Abstract

The aggregation of the islet amyloid polypeptide (IAPP) plays an important role in the pathology of type 2 diabetes (T2D). However, the transient and heterogeneous nature of the aggregated forms of IAPP makes it challenging to study their behaviour. In this study, we employed an antibody scanning approach by designing a panel of nine peptides targeting subsequent epitopes along the IAPP sequence. These peptides were then grafted into engineered single-domain antibody and monobody scaffolds, resulting in two panels of antibody-like constructs. We first tested these constructs for their ability to inhibit IAPP aggregation and assessed their binding affinity towards different IAPP species. Then, we utilized these constructs to detect IAPP species in serum samples obtained from T2D patients. This study illustrates the opportunities offered by the computational epitope scanning method to develop antibody-like constructs for the detection of IAPP aggregates in biological samples.

## Abstract 2

This study employs computational epitope scanning to design antibody-like constructs targeting IAPP, demonstrating their utility in inhibiting aggregation, binding IAPP species, and detecting pathogenic aggregates in type 2 diabetes serum samples.

## Graphical abstract

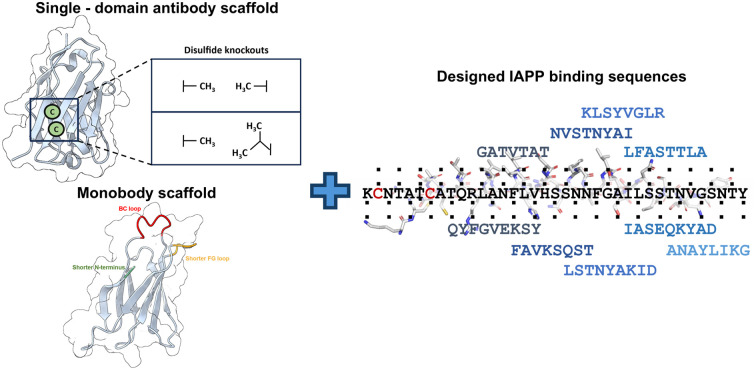

## Introduction

It has been estimated that nearly 500 million people – over 10% of the global adult population – suffer from diabetes as a result of aging populations and lifestyle factors^1–3^ with an annual cost close to $800 billion.^4^ Type 2 diabetes (T2D) accounts for 90% of cases, with late diagnoses in one-third of patients exacerbating complications and hindering treatment.^5,6^ While the precise causes remain unclear, this condition is strongly correlated with factors such as age, ethnicity, genetics, and obesity.^1–6^

T2D is characterized by hyperglycemia and insulin resistance, prompting an overproduction of two hormones, insulin and islet amyloid polypeptide (IAPP), which are co-expressed and co-secreted in the pancreas.^5,7–9^ Excessive IAPP production may result in its aggregation, a process that has been detected in over 90% of T2D patients and linked to β-cell dysfunction.^8,10,11^ The active form of IAPP comprises 37 amino acids, with an amidated C-terminus and an intramolecular disulfide bridge at the N-terminus.^8^ Similar to other misfolding proteins, previous research has revealed that the soluble prefibrillar aggregates, referred to as oligomers, play a pivotal role in driving cytotoxicity and are considered the most cytotoxic species generated during IAPP aggregation.^12–17^

The detection of small misfolded aggregates of amyloidogenic proteins is challenging.^18^ Enzyme-linked immunosorbent assay (ELISA) and western blot are frequently utilized for the measurement of IAPP levels in biological samples, and these detection techniques rely on the use of highly specific antibodies to capture the target protein.^16,17^ Most of these methods focus on detecting total IAPP species but lack specificity for aggregated forms. The small size of IAPP (over 10 times smaller than tau) and its rapid aggregation kinetics further complicate assay development. Therefore, developing antibodies for misfolding proteins like IAPP, and in particular antibodies that are selective for aggregated species, remains challenging.^19–23^

To address these challenges, we hypothesize that computationally designed antibody-like scaffolds can provide an effective solution for detecting and characterizing IAPP aggregates in T2D serum. Recent advances in experimental and computational methods are making it possible to design antibodies with *in silico* approaches, providing control over the target epitopes of the resulting antibodies.^20,24–26^ The success achieved in designing antibodies for aggregation-prone proteins, such as Aβ^22,27,28^ and α-synuclein,^29^ suggest that similar *in silico* approaches may be employed for the development of antibodies targeting IAPP.

Here, we engineered a single-domain antibody (sdAb) scaffold^30,31^ and a monobody scaffold^32,33^ to incorporate our computationally designed binding loops and support their structural and functional integrity, and then applied these scaffolds for the rational design of two panels of antibody-like protein binders scanning the sequence of IAPP. To design these antibody-like constructs, we employed a computational antibody scanning strategy based on the cascade method. This approach enables the generation of complementary paratope sequences that specifically target linear epitopes across the IAPP sequence. By grafting these sequences into structurally compatible scaffolds, we produced a panel of binders with diverse epitope specificities.^20,23^ After identifying binding candidates capable of differentiating monomeric and small aggregated IAPP species *in vitro*, we further utilized these candidates to quantify IAPP species in the serum of T2D patients using total internal reflection fluorescence (TIRF) microscopy^16,34^ and direct stochastic optical reconstruction microscopy (dSTORM).^35,36^

While the diagnosis of T2D can be made through established clinical markers, the role of IAPP aggregation in the progression and heterogeneity of the disease remains poorly understood. This study aims to provide a framework for detecting and characterising IAPP aggregates in serum, thereby enabling future investigations into how specific aggregate species may correlate with disease severity, β-cell health, or treatment outcomes.

## Results and discussion

### Improve sdAb scaffold by removing the conserved disulfide bond

We first evaluated whether the conserved disulfide bond in the sdAb scaffold could be removed prior to generating the DesAb library. The formation of this disulfide bond is challenging in the cytosolic expression in *E. coli*, and can hamper large-scale production, as disulfide bond formation requires an oxidative environment, whereas the prokaryotic cytosol typically maintains a reducing environment.^37^ Additionally, common affinity maturation techniques can result in incomplete formation of the conserved disulfide bond, potentially causing the exclusion of positive candidates during the selection.^38,39^ The presence of a population without disulfide bond can result in less active antibodies or lead to antibody aggregation, and therefore hamper the application of *in silico* approaches and display techniques for antibody development.^37,39,40^ Meanwhile, the heterogeneity arising from a population of non-disulfide antibodies poses a challenge to the reproducibility of these computational designed antibodies.^41^ Antibody-like protein scaffolds without intrinsic disulfide bonds can also serve as alternative scaffolds for computational design and offer unique advantages such as smaller size, higher stability and facile production in prokaryotic systems.^42,43^ The removal of the conserved disulfide bond may also facilitate the further chemical modification of sdAbs to introduce fluorophores, cytotoxic drugs or post-translational modifications (PTMs) as they usually require the introduction of an additional cysteine for site-specific modification.^44,45^ Although the removal of the disulfide bond through cysteine-to-alanine mutagenesis may lower the denaturation temperature of a nanobody, it may have no significant impact on the antigen binding affinity.^41^ Similarly, some single-chain variable fragment (scFv) variants could remain fully active when expressed in the cytoplasm without disulfide bonds.^46^ These results suggest that some sdAbs with disulfide bonds substituted may function normally in cytoplasm and may serve as intrabodies to bind to intracellular protein targets in various subcellular locations.

**Fig. 1:**
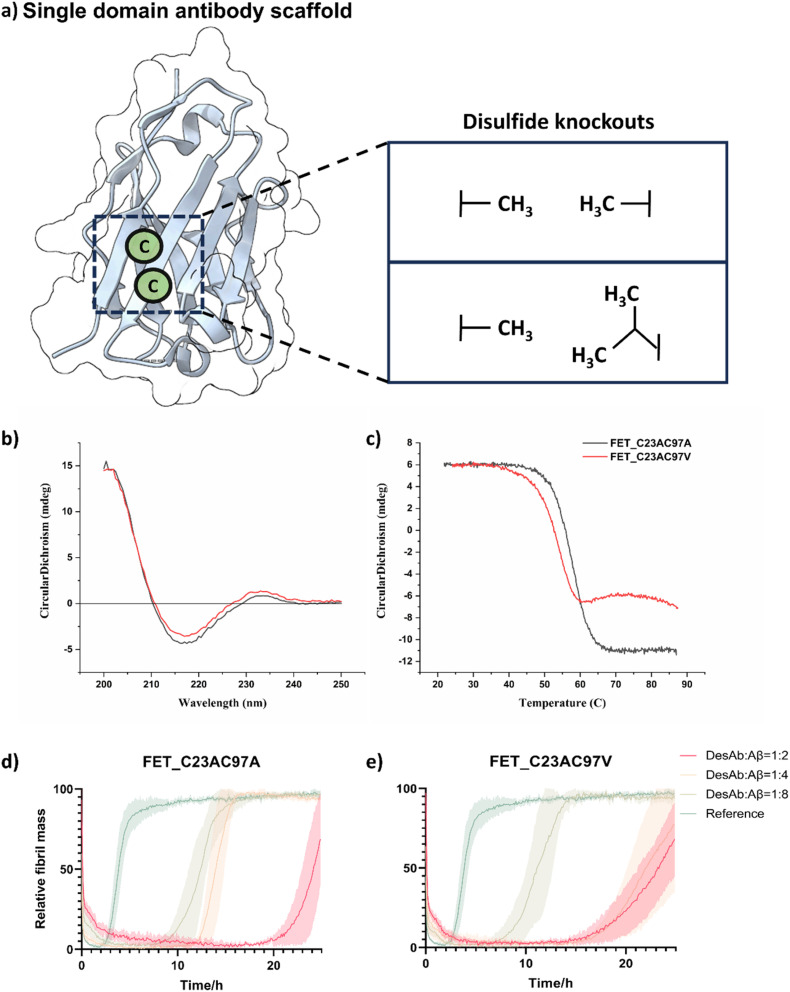
Removal of the conserved disulfide bond in the sdAb scaffold. (a) Schematic representation of the removal of the conserved disulfide bond in the sdAb scaffold. (b and c) Biophysical characterization of the 2 DesAbs obtained by mutating the cysteine residues at positions 23 and 97 (C23AC97A and C23AC97V) in the sdAb scaffold. Circular dichroism (CD) spectra (b) and CD thermal denaturation (c). All DesAb samples were measured at the concentration of 10 µM. (d and e) Aggregation assays of Aβ_42_ in the presence of the 2 DesAbs. The aggregation process of Aβ_42_ was monitored at 1 µM concentration in the presence of increasing concentrations of DesAbs in quadruplicate at 37 °C under quiescent conditions: DesAb : Aβ_42_ ratios 0 : 1 (as reference, green), 1 : 8 (light green), 1 : 4 (orange) and 1 : 2 (red). Both DesAbs exhibited a dose-depended delay of the aggregation process.

To eliminate the conserved disulfide bond in the sdAb scaffold, we tested two combinations of mutations (Fig. 1a and Scheme S1). The first combination involved replacing the conserved cysteine residues, Cys23 and Cys97, with alanine residues, as done previously.^41^ For the second combination, we substituted Cys23 with alanine and Cys97 with valine, an amino acid with a branched side chain that includes a second carbon atom, as previous research suggested that this substitution was better than the double-alanine in the context of scFvs.^47^

We further investigated whether disulfide removal would affect the binding and stability of the sdAb scaffold grafted with computationally designed complementary binding sequences. As a model for testing, we selected the previously characterised designed antibody (DesAb) DesAb-FETLTLR targeting Aβ_42_ (residues 3–9).^28^ Following expression and purification, we used circular dichroism (CD) spectroscopy to assess folding and thermal stability of these two constructs. CD revealed that these two constructs have a similar secondary structure content, fully compatible with that of well-folded single-domain antibodies from our previous research, including WT DesAb-FETLTLR (Fig. 1b and c).^28^ The thermal stability was decreased compared to the original sdAb scaffold. The melting temperatures were *T*_m_ = 59 °C for FETLTLR_C23A_C97A, *T*_m_ = 54 °C for FETLTLR_C23A_C97V, and *T*_m_ = 75 °C for WT DesAb-FETLTLR,^28^ which is consistent with previous observations.^41^ The melting curves suggest that FETLTLR_C23A_C97 V may experience some aggregation near 60 °C, as evidenced by a slight increase in the CD signal in this region. Conversely, FETLTLR_C23A_C97A and the previously reported WT DesAb-FETLTLR^28^ exhibited no such feature. Although the melting temperatures are higher than physiological conditions, we used thermal denaturation as a proxy for structural integrity and folding consistency of the constructs. This approach enabled us to prioritize variants that are more stable and homogeneous under experimental conditions, thus facilitating downstream applications such as labelling and imaging.

Next, we evaluated the inhibitory activity of FETLTLR_C23A_C97A and FETLTLR_C23A_C97V against Aβ_42_ aggregation through kinetic aggregation assays (Fig. 1d and e). The results indicated that both constructs were effective in inhibiting Aβ_42_ aggregation, as previously shown for the parent DesAb.^28^

Following these results, we selected FETLTLR_C23A_C97A for further analysis due to its higher thermal stability and neater melting signal, compared to FETLTLR_C23A_C97V. This choice was also supported by our experimental data (Fig. 1b–e) demonstrating that this combination retains the folding and binding activity of the scaffold while facilitating cytosolic expression and downstream chemical modifications. We tested the specificity of FETLTLR_C23A_C97A by performing a kinetics aggregation assay against α-synuclein (aS), a different misfolding protein. Since the FETLTLR sequence was designed for Aβ_42_, a specific DesAb should not inhibit the aggregation of aS, as indeed we found (Fig. S1).

### Engineering the monobody scaffold

Following the optimisation of the sdAb scaffold, we next investigated whether the monobody scaffold could also be adapted to support DesAb development. Antibody-like protein scaffolds offer several advantages that make them worth considering as framework for our computationally designed binding loops. First, their smaller size, ranging from 2 to 20 kDa, compared to the average 150 kDa for IgG antibodies, enables easier penetration into tissues and efficient filtration by the kidneys, which can be advantageous for certain applications.^48^ Second, these scaffolds tend to exhibit higher stability at elevated temperatures and can be produced in bacteria, yeast, and some even through chemical synthesis, eliminating the need for mammalian cell production. This makes the production process easier and more cost-effective.^42^

In this study, we selected a monobody as an additional scaffold for grafting designed binding loops due to its similar structure to the sdAb scaffold, which suggests that binding loops may be accommodated in a similar way. Compared to a sdAb a monobody has slightly smaller size, only two main binding loops (BC and FG loops), and it natively lacks cysteines in the scaffold, which simplifies expression and possible downstream chemical modifications.^32,33^

**Fig. 2:**
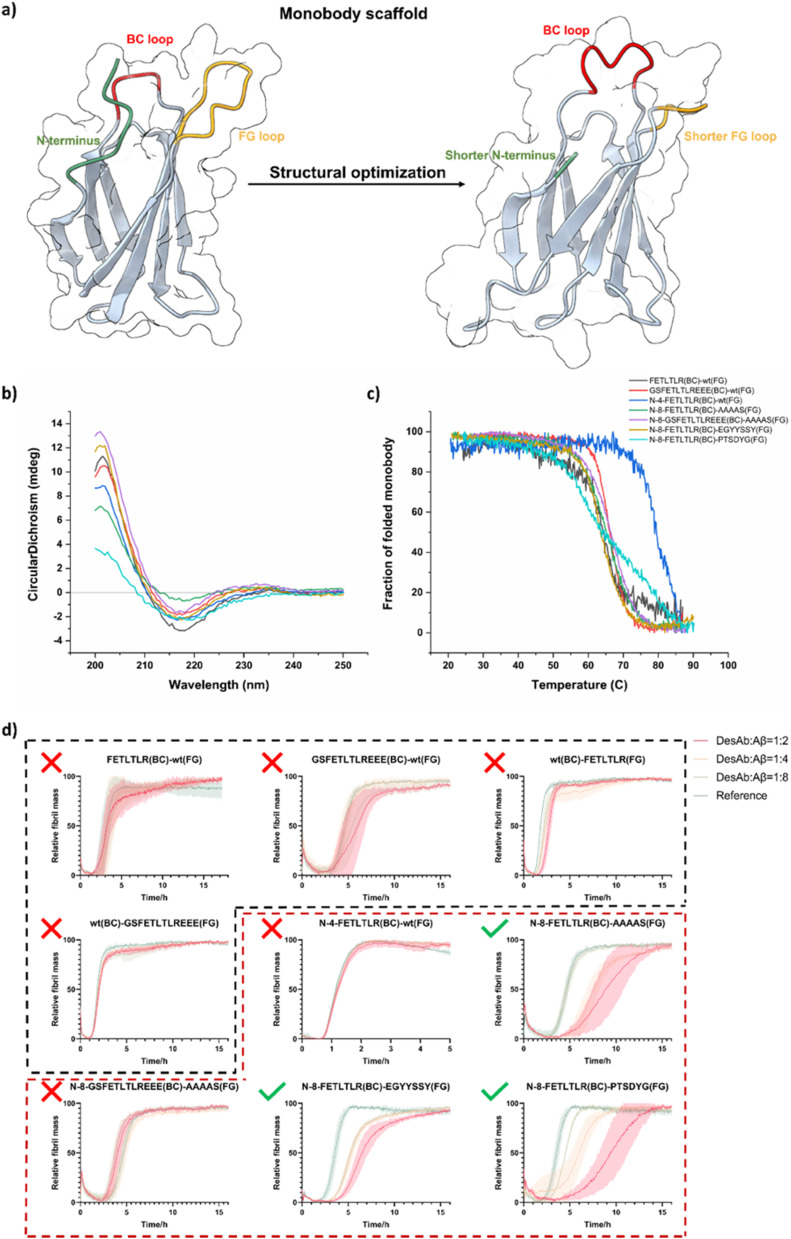
Engineering the monobody scaffold. (a) Schematic representation of the procedure used to engineer the monobody scaffold. (b and c) Biophysical characterization of different monobody variants. Circular dichroism (CD) spectra (b) and CD thermal denaturation (c). All monobody samples were measured at the concentration of 10 µM. (d) Aggregation assays of Aβ_42_ in the presence of the different monobody variants. The aggregation process of Aβ_42_ was monitored at 1.25 µM concentration in the presence of increasing concentrations of monobodies in quadruplicate at 37 °C under quiescent conditions. Monobody : Aβ_42_ ratios 0 : 1 (as reference, green), 1 : 8 (light green), 1 : 4 (orange) and 1 : 2 (red). The black dashed box indicates monobodies with the original scaffold and the red dashed box indicates monobodies with engineered scaffolds.

We used again the previously designed and validated FETLTLR sequence against Aβ_42_ (residues 3–9)^28^ for the grafting to the BC or FG loop of the monobody scaffold (Fig. 2a). We tested two grafting strategies: either just the designed sequence – FETLTLR or the whole CDR3 binding loop in the DesAb for Aβ_42_ – GSFETLTLREEE.^23,49^ In total, four monobody variants with the original monobody scaffold were generated: FETLTLR(BC)-wt(FG), GSFETLTLREEE(BC)-wt(FG), wt(BC)-FETLTLR(FG) and wt(BC)-GSFETLTLREEE(FG) (Schemes S2–S5).

After obtaining pure monobody variants, CD spectroscopy was used to detect the folding and thermal stability of these variants (Fig. 2b and c). We then tested their activity against the aggregation of Aβ_42_ using kinetics aggregation assays (Fig. 2d, black dashed box), and observed that all these monobody variants did not show significant activity against the aggregation of Aβ_42_. One possible explanation is that the N-terminal region, the designed loop, and the native loop may interfere with each other and impact binding *via* steric hindrance, as suggested by the structural models in Fig. 2a. To test for this possibility, we reduced the length of the N-terminal region and of the other WT loop, which led to an increased solvent exposure of the designed binding loop, thereby increasing its chance of interaction with the target protein. As the BC loop already possesses a sufficiently short length, we opted to shorten the FG loop and graft our designed sequence into the BC loop. By doing this, we generated three monobody variants with the engineered monobody scaffolds: N-4-FETLTLR(BC)-wt(FG), N-8-FETLTLR(BC)-AAAAS(FG) and N-8-GSFETLTLREEE(BC)-AAAAS(FG) (Schemes S6–S8). The N-terminal shortening was intended to improve BC-loop presentation and reduce local strain, whereas the FG-loop AAAAS substitution was used to lower steric bulk and increase loop permissiveness.

We then expressed and purified these monobody variants and used CD spectroscopy to assess their folding and thermal stability (Fig. 2b and c). Kinetics aggregation assays were applied to test their activity against the aggregation of Aβ_42_ under (Fig. 2d, red dashed box), and observed that only N-8-FETLTLR(BC)-AAAAS(FG) exhibited good activity against the aggregation of Aβ_42_, suggesting that simultaneous shortening of the N-terminus and FG loop may help maintain binding of the designed BC loop sequence. However, since activity recovery coincided with both modifications, causality cannot be assigned unambiguously. Because AAAAS is an artificial sequence and not ideal, we surveyed the sequences of monobody with reported structures (Fig. S2) and tested the two shortest sequences for FG loop and generated two monobody variants: N-8-FETLTLR(BC)-EGYYSSY(FG) and N-8-FETLTLR(BC)-PTSDYG(FG) (Schemes S9 and S10).

After obtaining these two new variants, we used CD spectroscopy to assess their folding and thermal stability (Fig. 2b and c). We then tested their activity against the aggregation of Aβ_42_ (Fig. 2d, red dashed box), and observed that both monobody variants showed good activity against the aggregation of Aβ_42_. The CD melting data of these two variants show similar melting temperatures, but rather different slopes, with N-8-FETLTLR(BC)-PTSDYG(FG) likely showing an earlier onset of unfolding (Fig. 2c). Therefore, we further measured their stability with nano differential scanning fluorimetry, which is sensitive to the tertiary structure content of the protein (Fig. S3). We found that N-8-FETLTLR(BC)-EGYYSSY(FG) exhibited a higher inflection temperature than N-8-FETLTLR(BC)-PTSDYG(FG), and therefore this was selected as the candidate monobody scaffold for the next set of experiments.

### Generation of a library of DesAbs to scan the sequence of IAPP

**Fig. 3:**
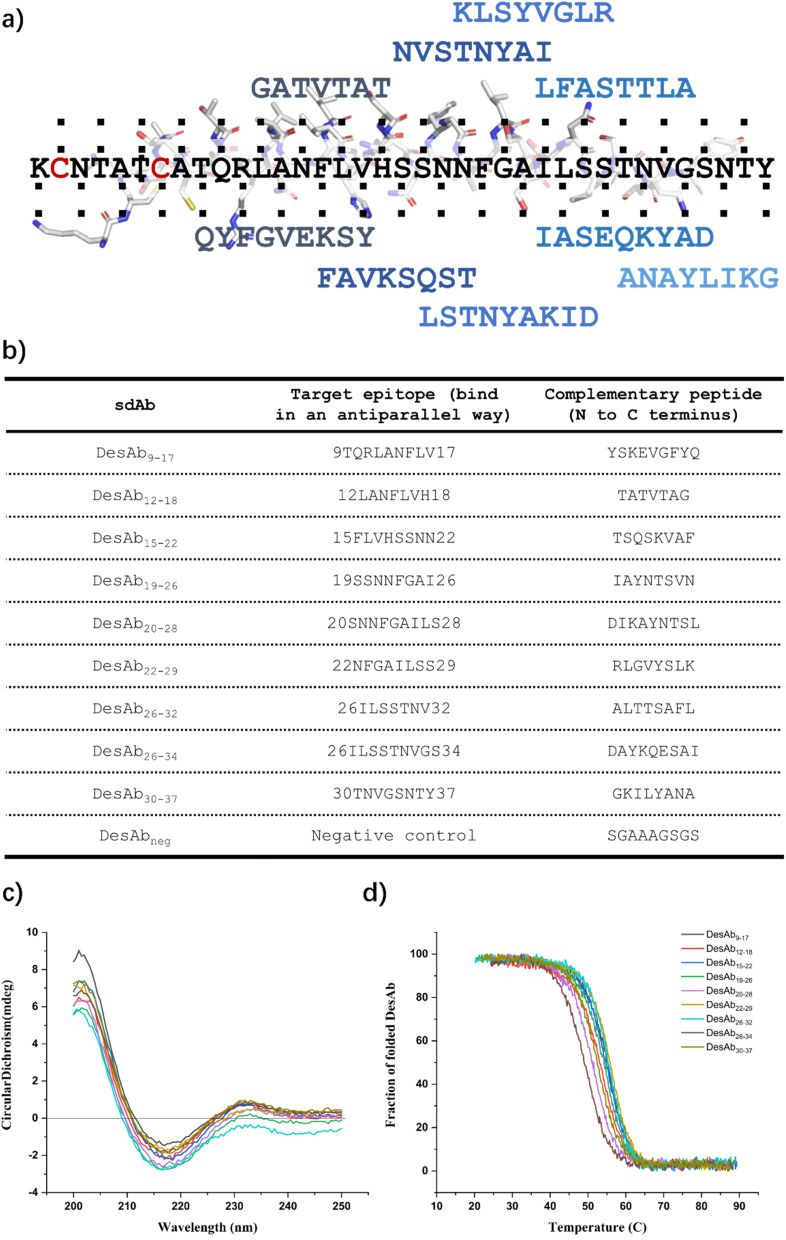
Generation of a panel of DesAbs to scan the sequence of IAPP. (a) Schematic representation of the 9 rationally-designed complementary peptides along the IAPP sequence. (b) List of generated antibodies, target sequences and grafted binding sequences. (c and d) Biophysical characterization of the 9 IAPP-targeting DesAbs. Circular dichroism (CD) spectra (c) and CD thermal denaturation (d). All DesAb samples were measured at the concentration of 10 µM.

We designed 9 peptides with the cascade method following the scaffold engineering,^23^ the workflow is summarised in the methods section. The 9 peptides scanned the whole sequence of IAPP, except for the N-terminal region, which includes the disulfide bond of Cys2 and Cys7 (Fig. 3a and b). In addition, we generated a negative control sequence composed solely of amino acids with short side chains (SGAAAGSGS), which was designed to not bind to any target. We then grafted these designed binding loops onto the CDR3 loop of the engineered sdAb scaffold with C23A and C97A substitutions (Scheme S11) to generate a library of DesAbs scanning the sequence of IAPP. These DesAbs were expressed and purified following (Materials and Methods). Expected folding (Fig. 3c and S4) and thermal stability (Fig. 3d and S4) were verified by CD.

**Fig. 4:**
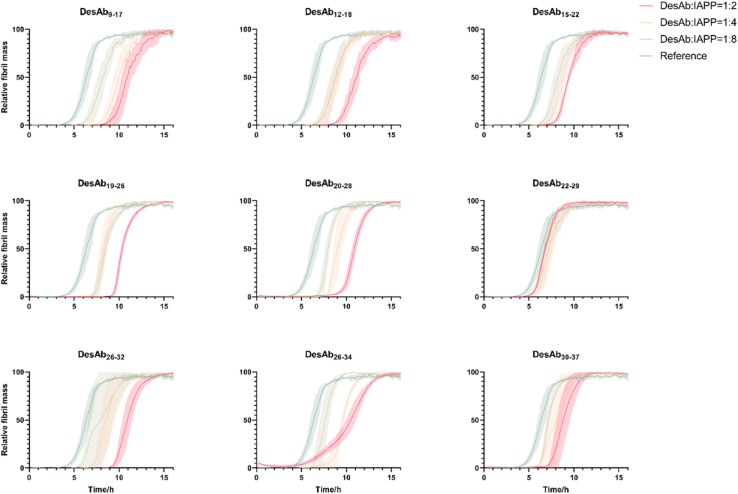
Aggregation assays of IAPP in the presence of the 9 DesAbs. The aggregation process of IAPP was monitored at 3 µM concentration in the presence of increasing concentrations of the DesAbs in quadruplicate at 37 °C under quiescent conditions. DesAb : IAPP ratios 0 : 1 (as reference, green), 1 : 8 (light green), 1 : 4 (orange) and 1 : 2 (red). All DesAbs except DesAb_22–29_ exhibited a dose-depended delay of the aggregation process.

To test the effects of these DesAbs on the aggregation of IAPP, the activity of DesAbs was measured in a kinetic aggregation assay of 3 µM IAPP under mildly acidic and quiescent conditions. Under these conditions, IAPP undergoes primary nucleation, elongation and surface-catalysed secondary nucleation.^50,51^ The formation of fibrils was then tracked by a fluorescent readout of the amyloid-specific dye ThT. We monitored the process of aggregation of IAPP at 3 µM concentration in the presence of increasing concentrations of the DesAbs (DesAb : IAPP ratios 0 : 1, 1 : 8, 1 : 4 and 1 : 2) in triplicate at 37 °C under quiescent conditions (Fig. 4). Our results indicated that all DesAbs except DesAb_22–29_ were effective in delaying IAPP aggregation in a concentration dependent manner, while the negative control DesAb did not show any activity against IAPP aggregation (Fig. S5). These results suggest although DesAb_22–29_ displays measurable affinity for both monomeric and fibrillar IAPP (Tables 1 and S2), it acts as a bystander binder, as it binds the reactants and the products in regions that do not perturb the rate-determining steps of self-assembly.

**Table 1:** Binding affinity of the DesAbs reported in this work against monomeric and fibrillar IAPP^a^

DesAb	Monomers *K*_D_ (µM)	Fibrils *K*_D_ (µM)
DesAb_9–17_	2.07	ND
DesAb_12–18_	ND	ND
DesAb_15–22_	503	2.08
DesAb_19–26_	0.400	ND
DesAb_20–28_	0.201	ND
DesAb_22–29_	0.529	1.87
DesAb_26–32_	1.40	ND
DesAb_26–34_	0.324	1.12
DesAb_30–37_	0.410	ND
DesAb_neg_	ND	ND

a ND: not detected.

To determine the binding affinity (*K*_D_) to monomeric IAPP, we used bio-layer interferometry (BLI) with immobilized biotin-IAPP monomers, obtaining *K*_D_ values with varying concentrations of DesAbs. Most of the measurements generated *K*_D_ values in the hundreds nM to low µM range (Tables 1, S2 and Fig. S6), which are comparable to those found for DesAbs designed to bind Aβ_42_.^22,27^ We also measured the binding affinity of DesAb_neg_ as negative control, and it did not show binding signal to IAPP monomers (Fig. S7).

The binding to IAPP fibrils was determined by BLI by immobilizing pre-prepared IAPP fibrils onto AR2G biosensors. Only 3 DesAbs (DesAb_15–22_, DesAb_22–29_ and DesAb_26–34_) showed binding signal to IAPP fibrils with low µM *K*_D_ at DesAb concentrations between 1 and 6 µM (Tables 1, S2 and Fig. S8). The lack of binding signal to IAPP fibrils, combined with the observed inhibition of the aggregation and the presence of binding to the monomer, suggest that some of the epitopes are inaccessible to the DesAbs in a fibrillar conformation, at least in the BLI setup that we used here. We also measured the binding of DesAb_neg_ as negative control, and it did not show binding signal to IAPP fibrils (Fig. S9). We note that although DesAb_12–18_ produced no measurable response with either monomers or mature fibrils in the BLI measurements (Fig. S9), it nonetheless caused a delay in the overall aggregation reaction (Fig. 4). These findings can be explained by the species that each method can see. BLI is used for the stable endpoints of the reaction, *i.e.* soluble monomers and surface-immobilised fibrils, because these can be readily covalently tethered to the sensor. The transient, on-pathway oligomers that dominate the early stages of aggregation are therefore not visible in the assay. Our data thus suggest that DesAb_12–18_ likely recognises an epitope uniquely exposed during the formation of on-pathway oligomers.

### Generation of a library of monobodies against IAPP

**Fig. 5:**
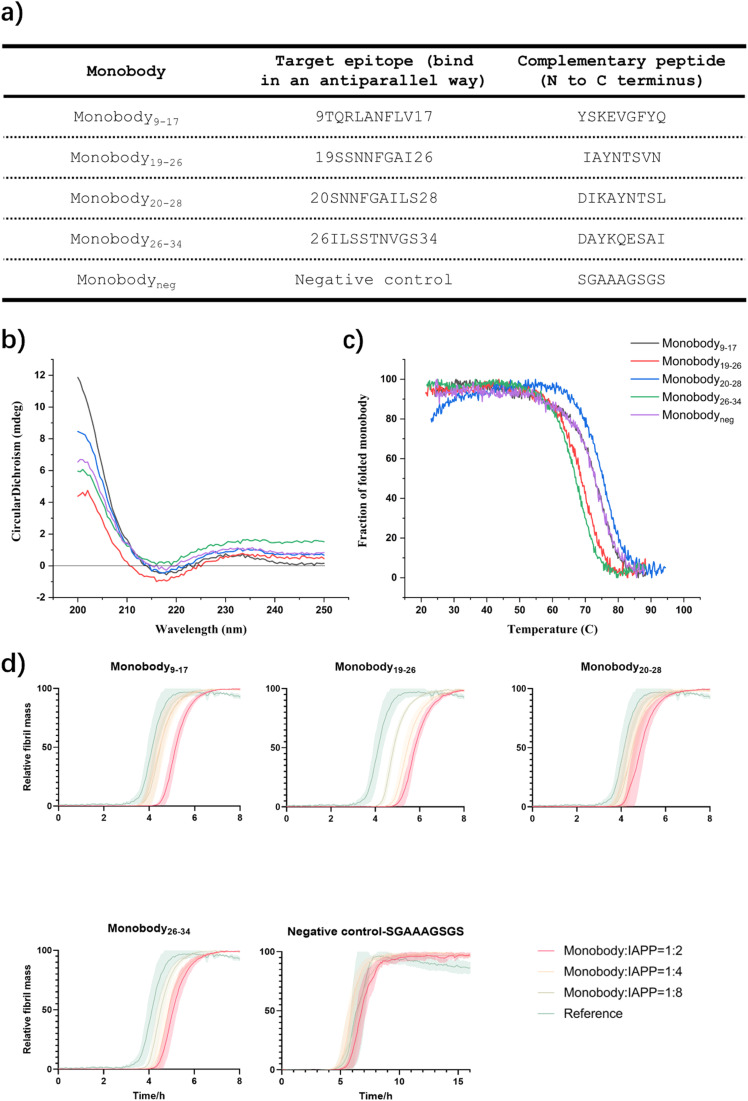
Generation of a panel of monobodies to scan the sequence of IAPP. (a) List of generated monobodies, target sequences and grafted binding sequences. (b and c) Biophysical characterization of the generated IAPP-targeting monobodies. Circular dichroism (CD) spectra (b) and CD thermal denaturation (c). All monobody samples were measured at the concentration of 10 µM. (d) Unseeded kinetic aggregation assays of the designed monobodies against IAPP. The aggregation process of IAPP was monitored at 3 µM concentration in the presence of increasing concentrations of the monobodies in quadruplicate at 37 °C under quiescent conditions. Monobody : IAPP ratios 0 : 1 (as reference, green), 1 : 8 (light green), 1 : 4 (orange) and 1 : 2 (red). All monobodies with designed IAPP-targeting sequences exhibited a dose-dependent delay in the aggregation process, which was not observed in the negative control.

We selected several of the most promising paratopes (Fig. 5a) that were confirmed using the sdAb scaffold described above, and grafted them onto the BC loop of our engineered monobody scaffold (Scheme S12) to generate a library of monobodies against IAPP. The selection criteria are based on binding affinity to monomer using BLI, aggregation inhibition and imaging capability using TIRF microscopy (Table S2). Each monobody variant was expressed and purified (Materials and Methods), and their correct folding (Fig. 5b) and thermal stability (Fig. 5c) were verified by CD.

We measured the activity of these monobodies in a kinetic aggregation assay of 3 µM IAPP under the same mildly acidic and quiescent conditions we used for DesAbs. The formation of fibrils was then tracked by a fluorescent readout of the amyloid-specific dye ThT. We monitored the process of aggregation of IAPP at 3 µM concentration in the presence of increasing concentrations of the monobodies (monobody : IAPP ratios 0 : 1, 1 : 8, 1 : 4 and 1 : 2) in triplicate at 37 °C under quiescent conditions (Fig. 5d). Our findings demonstrate that all monobodies were capable of delaying IAPP aggregation in a concentration-dependent manner while the negative control monobody did not show activity against IAPP aggregation (Fig. 5d). However, their efficacy was comparatively lower than that of their DesAb counterparts.

We then applied BLI to determine the binding affinity of these monobodies to monomeric IAPP or IAPP fibrils by using the same concentration and condition for DesAbs, but we did not observe obvious binding signal for these monobodies. We concluded that these IAPP monobodies have relative low binding affinity to monomeric IAPP or IAPP fibrils compared to their DesAb counterparts.

### Diffraction-limited TIRF imaging of IAPP monomers and small aggregates

**Fig. 6:**
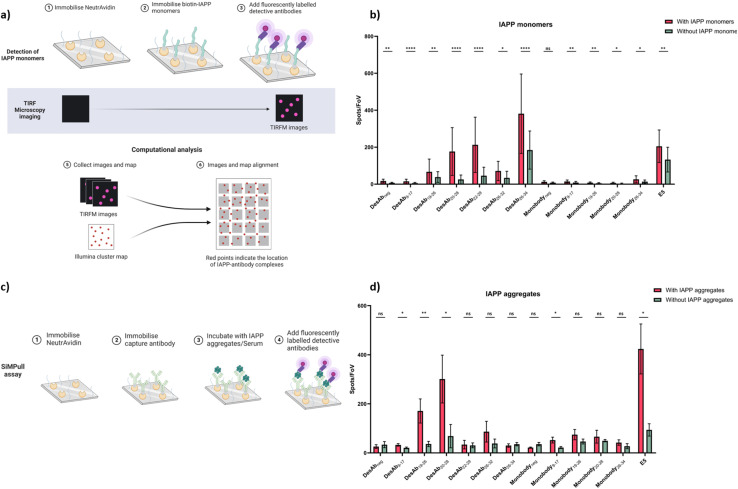
TIRF detection of IAPP monomers and aggregates. (a) Schematic representation of the set-up of TIRF microscopy for the *in vitro* detection of IAPP monomers. (b) Detected spots per field of view for IAPP monomers under TIRF microscopy by using different fluorescence-labelled antibodies. (c) Schematic representation of the set-up of TIRF microscopy for SiMPull assays to detect IAPP aggregates with pre-prepared IAPP aggregates or in serum samples of T2D patients. (d) Detected spots per field of view for pre-prepared IAPP aggregates under TIRF microscopy by using a commercial antibody-E5 as capture antibody and different fluorescence-labelled designed antibodies as detection antibodies.

According to their binding affinity and activity against IAPP aggregation, seven of the DesAbs (DesAb_9–17_, DesAb_19–26_, DesAb_20–28_, DesAb_22–29_, DesAb_26–32_, DesAb_26–34_ and DesAb_neg_) and five monobodies (Monobody_9–17_, Monobody_19–26_, Monobody_20–28_, Monobody_26–34_ and Monobody_neg_) were labelled with Alexa Fluor™ 647 by *N*-hydroxysuccinimide (NHS) ester chemical conjugation to primary amines. We used the commercial monoclonal anti-IAPP antibody E5, labelled with Alexa Fluor™ 647, as the positive control. Biotin-IAPP monomers were immobilised on the coverslip through the pre-immobilised NeutrAvidin (Fig. 6a). The improved imaging surface used in this work is based on the self-assembly of F127 on glass pre-coated with Rain-X, a readily available household chemical. In contrast to traditional covalent chemistry-based coatings, this approach is far simpler, making the platform more accessible to laboratories with limited chemistry facilities. We reconstituted monomeric IAPP with PBS spiked with human serum albumin (HSA) to prevent further aggregation^52,53^ Although we used BLI to determine binding affinities to purified IAPP monomers and fibrils (Tables 1, S2, Fig. S6 and S7), the complexity of serum samples and the need for morphological resolution motivated our use of TIRF and dSTORM imaging. These microscopy techniques provide single-particle sensitivity and allow the spatial characterisation of IAPP species in a way that is not achievable with bulk methods such as BLI.

The binding signals of the designed antibody-like scaffolds were compared with their corresponding blanks (without immobilised biotin-IAPP) and the fluorophore-labelled negative control DesAb_neg_/Monobody_neg_ (Fig. 6b, S10 and S11). Several DesAbs demonstrated enhanced binding signals relative to their corresponding blanks, notably DesAb_9–17_, DesAb_19–26_, DesAb_20–28_, DesAb_22–29_, DesAb_26–34_, along with two monobodies, Monobody_9–17_ and Monobody_19–26_, which showed similar or greater significance compared to DesAb_neg_. When comparing with the commercial monoclonal anti-IAPP antibody E5, five DesAbs (DesAb_9–17_, DesAb_19–26_, DesAb_20–28_, DesAb_22–29_ and DesAb_26–34_) exhibited similar or better binding to IAPP monomers.

To test the detection of IAPP small aggregates by the designed antibody-like scaffolds, a single-molecule pull-down (SiMPull) assay was used in combination with TIRF microscopy (Fig. 6c). We used E5 as the capturing antibody to be immobilized on the coverslip. IAPP aggregates were prepared (Fig. S12) as described in Materials and Methods and incubated with the capture antibody. After several blocking and washing steps, IAPP aggregates were detected with fluorophore-labelled antibodies (Fig. 6d, S13 and S14). The results are shown in comparison to their corresponding negative control antibodies (DesAb_neg_ and Monobody_neg_) and the fluorophore-labelled E5. Data were obtained by averaging over the intensity of 40 stack images at one field of view. For each sample we did three repeats, and each repeat contains 16 different fields of view. DesAb_9–17_, DesAb_19–26_, DesAb_20–28_, and Monobody_9–17_ exhibited a significant increase in signal compared to their corresponding blank control. While the overall fluorescence counts for DesAb_neg_ are around 10-fold less than DesAb_20–28_, they were slightly higher than the buffer-only control (Fig. 6d). We attribute this small offset to stronger DesAb_neg_–E5 interaction, as well as the marginally higher degree of Alexa Fluor 647 labelling on DesAb_neg_ (Table S3), which could increase its nonspecific adsorption to the PEG-coated surface. The commercial antibody E5 also exhibited a strong binding signal to IAPP aggregates. We further assessed antibody specificity by calculating the ratio of signals between positive and negative samples. DesAb_20–28_ showed greater sensitivity than E5 for both monomeric and aggregated species, whereas DesAb_19–26_ exhibited a comparable ratio to E5 (Fig. S18).

We also note that in the BLI assay, fibrils are covalently immobilised on AR2G sensors through amine coupling, which partially buries internal β-strand surfaces and therefore masks lateral epitopes. By contrast, in SiMPull the aggregates are first captured by the anti-IAPP antibody E5 *via* the N-terminal epitope, leaving the fibril sides fully accessible. Consequently, DesAb_19–26_ recognises an epitope that is likely exposed in the SiMPull geometry, but partly occluded in the BLI geometry, whereas DesAb_22–29_ targets a region that remains exposed in both formats. We emphasise that the apparent *K*_D_ of DesAb_19–26_ for fibrils likely lies below the sensitivity limit of our BLI set-up, and yet the multivalent binding on the high local-density SiMPull surface is sufficient for detection.

### dSTORM imaging of IAPP monomers and small aggregates

**Fig. 7:**
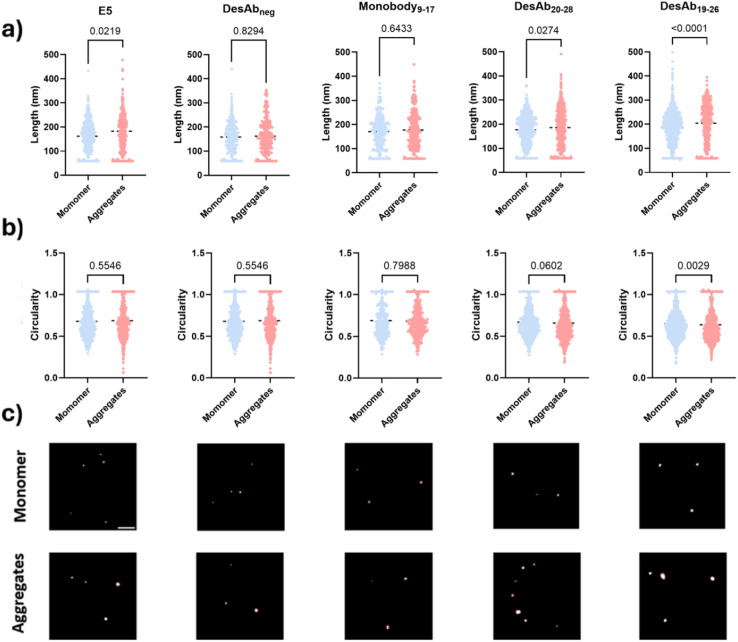
Super-resolution characterisation of small IAPP monomers and small aggregates. IAPP species were immobilised onto the surface *via* the E5 antibody and visualised with the selected fluorescent designed antibody-like scaffolds (DesAb_neg,_ Monobody_9–17,_ DesAb_20–28,_ DesAb_19–26_) and E5 as positive control. (a) Size comparison between IAPP monomers and small aggregates using dSTORM imaging using selected fluorescently-labelled designed antibody-like scaffolds. Size information was quantified by the Maximal Feret parameter (nm). (b) Shape comparison between IAPP monomers and small aggregates using dSTORM imaging. Shape information was quantified by circularity. (c) Representative super-resolution images. Scale bar = 1 µm.

We further assessed whether the designed antibody-like scaffolds could detect IAPP monomers and small aggregates using super-resolution microscopy by dSTORM imaging (Fig. 7). The fluorescently-labelled designed antibody-like scaffolds were utilised to super-resolve IAPP monomers and aggregates. DesAb_19–26_, DesAb_20–28_, and Monobody_9–17_ were selected for testing because of their higher signal compared to their blank controls. E5 and DesAb_neg_ were also included as positive and negative controls, respectively. We found that DesAb_19–26_ and DesAb_20–28_ could distinguish the size difference between IAPP monomers and small aggregates (Fig. 7a) while DesAb_19–26_ identified a shape difference between IAPP monomers and small aggregates (Fig. 7b). The similarity in circularity between monomeric and aggregated IAPP species likely arises from the globular morphology of small early-stage aggregates, combined with resolution limitations inherent to dSTORM imaging at this size scale. These aggregates are not yet fibrillar, and their dimensions and fluorescent labelling profiles can result in shapes with circularity values similar to those of monomers. The average length of monomeric IAPP measured by E5, DesAb_neg_, Monobody_9–17_, DesAb_20–28_ and DesAb_19–26_ were 162 nm, 159 nm, 171 nm, 177 nm and 187 nm, respectively, while for aggregates, they were 183 nm, 162 nm, 177 nm and 186 nm, and 204 nm respectively. We note that E5, the positive control, could not distinguishing with statistical significance the shape differences between IAPP species, likely because the larger size of antibody can induce more significant size exclusion and therefore disadvantageous while being used as probes in super-resolution microscopy. We also compared the size measured from single DesAb and IAPP species to ensure the detection of aggregated species (Fig. S17).

### Diffraction-limited TIRF detection of IAPP species in T2D serum

**Fig. 8:**
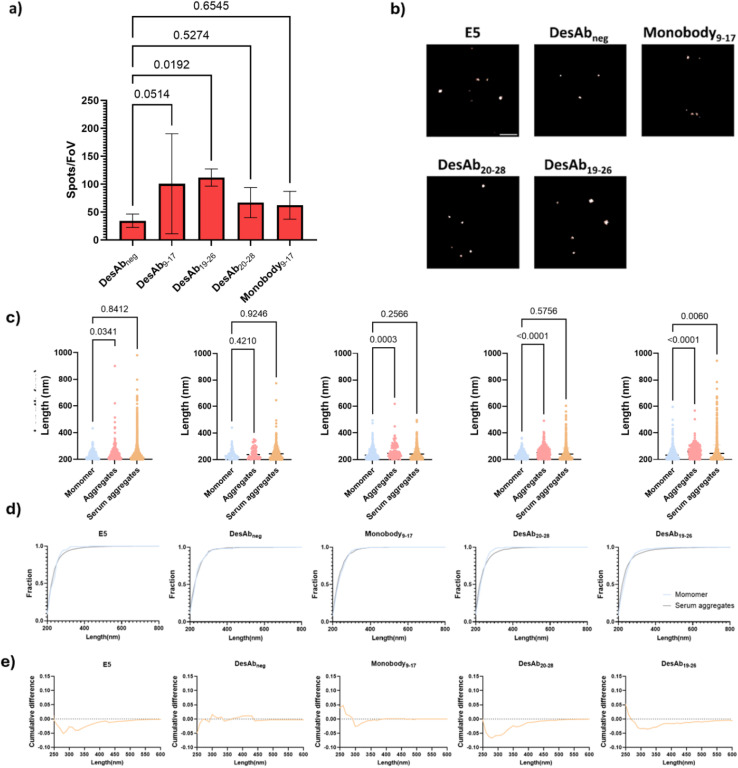
Detection of IAPP aggregates in serum samples of T2D patients with fluorescence microscopy. (a) Detected spots per field of view for IAPP species in serum samples under TIRF microscopy by using E5 as capture antibody and different fluorescence-labelled designed antibody-like scaffolds as detection antibodies. (b) Representative super-resolution images of IAPP species in human T2D serum. Scale bar = 1 µm. (c). Size distribution of larger IAPP species (>200 nm) detected by different antibodies in serum. (d). Cumulative histogram of larger IAPP species (>200 nm) detected by different antibodies in serum. (e) Cumulative difference of larger IAPP species (>200 nm) detected by different antibodies in serum, showing that large (300–400 nm) IAPP aggregates can be detected in serum.

We then applied the SiMPull assays to detect IAPP aggregates in the T2D serum under TIRF microscopy (Fig. 6c). E5 was immobilized on the coverslip to act as capture antibody. T2D serum samples from six patients were used for this experiment (Table S1). After incubation with the T2D serum and several blocking and washing steps, IAPP species were detected to different extents with the fluorescently-labelled designed antibody-like scaffolds (Fig. 8a, S15, S16 and S19). DesAbs_19–26_ produced significantly higher signals than DesAbs_neg_, indicating that it can specifically detect protein aggregates in complex samples. Moreover, DesAb_9–17_ and E5 displayed no significant differences relative to their corresponding blank controls, attributable to the high variance within the experimental group and the notably high background signal associated with E5 (Fig. S21). We observed that approximately 30% of serum samples produced strong signals with the IgG isotype control, whereas no such reactivity was detected with DesAbs. This indicates that DesAbs are less prone to cross-talk with heterophilic antibodies in complex human biofluids, highlighting their potential as reliable tools for imaging and related applications (Fig. S19).

### dSTORM detection of IAPP aggregates in T2D serum

We further super-resolved the IAPP species in T2D serum to confirm the detection of IAPP aggregates in serum. We observed a tailed size distribution for IAPP species in T2D serum but not in monomeric IAPP (Fig. 8c). The cumulative histograms (Fig. 8d and e) showed that DesAb_20–28,_ DesAb_19–26_ and E5 were able to detect molecules with size of ∼300 nm in serum but barely in monomeric IAPP. Statistical analysis further demonstrated that the distribution of IAPP species visualised by DesAbs_19–26_ differs significantly from monomers, indicating that it specifically detects aggregated IAPP. This is likely due to its higher binding affinity, which enables sufficient engagement with individual molecules for morphological mapping, consistent with the single-molecule counting data. Notably, these larger serum-derived species comprised less than 2% of total aggregates, and they are undetectable with Monobody_9–17_ and DesAb_neg_.

## Conclusions

We reported the rational design of two panels of antibody-like scaffolds to scan the sequence of IAPP. Our results indicated that this approach can lead to the generation of antibody-like scaffolds capable of detecting IAPP species in T2D serum. By using super-resolution microscopy, we showed that the quantification of heterogeneous IAPP species in human serum enables the characterisation of their morphological properties. Compared to the traditional SiMPull platform,^29^ the enhanced imaging surface used in this study significantly reduces both the cost and preparation time for imaging. This simple and user-friendly platform is particularly suitable for physics or biology-focused laboratories with limited access to specialised chemistry facilities. Additionally, this research pipeline is compatible with high-throughput systems supported by automated liquid handling, facilitating further high-throughput antibody screening.^61^ Overall, our approach illustrates the potential of the antibody scanning method to develop antibody-like scaffolds for the detection and quantification of IAPP aggregates in biological samples. By distinguishing IAPP species in human serum, this approach offers a route to investigate the relationship between IAPP aggregation and the heterogeneity of T2D, potentially aiding biomarker discovery and patient stratification in future studies.

## Materials and Methods

### Computational design

We employed the cascade method for paratope generation due to its established ability to design aggregation-inhibiting binders by targeting β-sheet interfaces with high specificity.^20,23^ This method offers the advantage of generating structurally compatible and soluble sequences suitable for grafting into antibody-like scaffolds. In brief, the complementary peptides are built through a fragment-based procedure. The first step is the identification of short peptide fragments that interact in a β-strand conformation with short peptide fragments of the target sequence in at least one of the protein structures in the PDB database. The short peptide fragments are then linked together to form a complementary peptide (paratope) for the given epitope. We first generated potential candidates with different hydrogen binding patterns to target the N-terminus of Aβ_42_ (residues 3–9, which covers the epitope of aducanumab (residues 3–7),^54^ the therapeutic antibody from Biogen Inc. that was recently approved for the treatment of AD), and then selected those with high complementarity score C and CamSol score. Our parent antibody (DesAb-HETLTLR, Fig. S2.5.4) was previously shown to be able to inhibit both primary and secondary nucleation of Aβ_42_ and decrease the formation of oligomers.^27^ The resulting paratope candidates were then grafted into compatible loops of antibody-like scaffolds (here, sdAb and monobody scaffolds). Structural models generated by AlphaFold or ESMFold were used to verify loop conformations and minimise steric clashes with the scaffold. Circular dichroism spectroscopy was applied to assess folding and stability of the constructs, while aggregation assays and bio-layer interferometry (BLI) were used to evaluate their activity.

### Circular dichroism

Far-ultraviolet (UV) circular dichroism (CD) spectroscopy was used to analyze the secondary structure of the DesAbs in solution, as recorded by a Jasco J-810 spectropolarimeter equipped with an Applied Photophysics Chirascan system and a Quantum TC125 temperature control unit, using a 0.1 cm pathlength cuvette. Samples contained 10 µM protein in 20 mM sodium phosphate (NaP) and 100 mM sodium chloride buffer at pH 8. The far-UV CD spectra of all DesAbs were recorded from 200 to 250 nm at 25 °C and averaged over three scans, and the spectrum of the buffer was subtracted from the averaged data. All spectra were measured as *θ* (in *m*deg.).

### Thermal stability assay

The thermal stability of the DesAbs was analyzed by monitoring the CD signal at 207 nm from 25 to 90 °C at a rate of 0.5 °C min^−1^. Data points were acquired every 0.1 °C with a bandwidth of 1 nm. Analysis of the thermal unfolding curves was performed, assuming a two-state unfolding model.

### Aβ_42_ expression and purification

Aβ_42_ peptides were expressed in *E. coli* BL21 (DE3) pLysS cells (Agilent Technologies) and extracted, as described previously.^55^ Before running kinetics experiments, the purified lyophilized Aβ_42_ peptide was dissolved in 6 M guanidine hydrochloride (GuHCl) (pH 8) and incubated for at least 45 min on ice. This solution was then subjected to gel filtration using a Superdex 75 10/300 GL column on an Äkta Pure system (GE Healthcare), and the peak corresponding to the monomeric Aβ_42_ peptide was collected in low-binding test tubes (Corning) on ice. The column was equilibrated with 20 mM NaP buffer supplemented with 200 µM ethylenediaminetetraacetic acid (EDTA) at pH 8.

### aS expression and purification

We followed a previously published protocol.^56^ In brief, *E. coli* BL21(DE3) cells were transformed with pT7-7 αS and cultured in a LB medium. Protein expression was induced by 1  IPTG for 4 h at 37 °C. Bacteria were harvested, and pellets were lysed in 10 mM Tris(hydroxymethyl)aminomethane hydrochloride (Tris–HCl) (pH 8.0), 1 mM EDTA, 1 mM phenylmethylsulfonyl fluoride (PMSF). The lysate was sonicated for 5 min and boiled subsequently for 15 min, followed by centrifugation. The supernatant was subjected to streptomycin sulfate and ammonium sulfate precipitation steps as described. The ammonium sulfate pellet formed after centrifugation at 5200×*g* for 30 min was dissolved in 50 mM Tris–HCl (pH 7.5), 150 mM KCl and subjected to SEC on a Superdex 200 column (GE Healthcare, Chalfont St Giles, UK). Aliquots were flash-frozen in liquid N_2_ and stored at −80 °C.

### Preparation of aS seeds

Monomeric aS was buffer-exchanged to MES buffer (10 mM 2-(*N*-morpholino) ethanesulfonic acid, 1 mM EDTA, pH 5.5) and concentrated to 200–300 µM using 10 kDa molecular weight cut-off (MWCO) centrifugal spin filters (Amicon Ultra, Millipore). Concentrated aS monomers were incubated in protein low-binding tubes (Eppendorf) for 72 h at 40 °C and 400 rpm with a magnetic stirrer. In a benchtop centrifuge, the solution was centrifuged at 21 130×*g* to determine the fibril concentration (monomeric equivalent) (Eppendorf). By measuring absorbance with a NanoDrop 2000 (Thermo Scientific), the concentration of the remaining aS monomer detected in the supernatant was calculated and subtracted from the initial concentration. After MES buffer was added to the supernatant's volume, the stock was aliquoted and kept at −80 °C. The fibril stock was diluted to a final concentration of 5 µM in protein low binding tubes and sonicated for 15 seconds to create aS seeds.^29^

### DesAbs expression and purification

Genes encoding the two groups of DesAbs were generated through site directed mutagenesis from the gene of the WT DesAb-HETLTLR (Fig. S2.5.4) in a pET28 plasmid. The different antibodies were expressed in *E. coli* SHuffle T7 LysY (NEB) competent cells (Table S2.5.1). Cells were plated and starter cultures inoculated from single colonies were grown overnight for 15 h at 37 °C at 200 rpm in lysogenic broth (LB) medium supplemented with kanamycin (50 µg mL^−1^). Glycerol stocks were made by taking 700 µL of the starter cells supplemented with 700 µL glycerol, and stored at −80 °C. Growth was started in 1 L LB medium supplemented with kanamycin (50 µg mL^−1^) at 26 °C for about 8 h (to OD_600_ = 0.6–0.8), the temperature was then lowered to 20 °C and isopropyl β-d-1-thiogalactopyranoside (IPTG) was added to a final concentration of 0.2 mM. After overnight expression at 20 °C, cells were harvested by centrifugation at 7500 rpm (JA-8.1 rotor, Beckmann Coulter) and resuspended in 45 mL 20 mM sodium phosphate (pH 8.0) buffer supplemented with 10 mM imidazole and one tablet of Roche Complete EDTA-free protease inhibitor cocktail. Cells were then lysed using sonication for 5 min at 40% amplitude in 15 s on and 45 s off cycles. The supernatant containing the protein was separated from cell debris using centrifugation at 18 000 rpm (JA-20 rotor, Beckmann Coulter). The cleared lysate was loaded onto a Ni^2+^-NTA Superflow column (Qiagen), equilibrated with 20 mM sodium phosphate buffer (pH 8.0) containing 10 mM imidazole. Non-specifically bound proteins were then washed away with 100 mL of 20 mM NaP buffer containing 60 mM imidazole, and the his-tagged DesAb was eluted with 20 mM NaP buffer containing 200 mM imidazole. The purity of the collected fractions was assessed by SDS-PAGE, and only the purest fractions were collected and dialysed in 20 mM NaP 100 mM NaCl buffer for 20 h, and finally concentrated to a concentration between 10 µM and 30 µM *via* an Amicon Ultra – 15/10k *M*_w_. cut-off filter (Millipore), flash frozen and stored in the −80 °C.

### Aggregation assay for Aβ_42_

Solutions were prepared containing monomeric 1 µM Aβ_42_ peptide in the presence of increasing amounts of each DesAb (90% v/v of 1 µM Aβ_42_ peptide and 10% v/v of DesAbs, final buffer would be 20 mM NaP 10 mM NaCl 180 µM EDTA, supplemented with 20 µM ThT). Seeded experiments were performed in the presence of 40% preformed fibrils that were prepared freshly the same day^57^ with increasing antibody-to-Aβ_42_ monomer ratio (0 : 1, 1 : 8, 1 : 4 and 1 : 2). Unseeded kinetic aggregation assays of the DesAb-HETLTLR(WT) and DesAb-FETLTLR were also performed with and without the presence of 1% w/v human cerebrovascular disease brain homogenate prepared as previously described.^58^ Each sample was then pipetted into multiple wells of a low-binding 96-well half-area plate of black polystyrene with a clear bottom and polyethylene glycol coating (Corning 3881) (80 µl per well). Plates were sealed to prevent evaporation. Aggregation assays were performed at 37 °C under quiescent conditions using a CLARIO or OMEGA star plate reader (BMG Labtech). The concentration of Aβ_42_ (1.25 µM) and IAPP (3 µM) was chosen based on their respective aggregation behaviours, with IAPP requiring a higher concentration to achieve consistent and measurable aggregation kinetics under our assay conditions. The ThT fluorescence was measured through the bottom of the plate every minute with an excitation filter of 440 nm and an emission filter of 480 nm.

### Aggregation assay for aS

A Superdex 75 10/300 GL column (GE Healthcare) was used to gel filter purified aS while it was equilibrated in MES buffer (10 mM 2-(*N*-morpholino) ethanesulfonic acid, 1 mM EDTA, pH 5.5). The peak corresponding to monomeric aS peptide was then collected in a low-binding test tube (Corning) on ice. Monomeric aS peptides were used to prepare solutions at a protein concentration of 40 µM in the presence of increasing amounts of the specified sdAb/monobody variant in MES buffer supplemented with 5 µM ThT and 0.5% α-synuclein seeds. Then, 150 µl of sample was pipetted into each well of a 96-well half-area plate of black polystyrene with a clear bottom and polyethylene glycol coating (Corning). Evaporation was prevented by sealing the plates. Utilizing a CLARIOstar plate reader (BMG Labtech), aggregation experiments were carried out at 37 °C in quiescent conditions. With a 440 nm excitation filter, the ThT fluorescence was monitored through the plate's bottom at 480 nm every minute.^29,59^

### DesAbs expression and purification

Genes encoding the conserved disulfide bond removed DesAbs were generated through site directed mutagenesis from the gene of the DesAb-FETLTLR in a pET28 plasmid. The different antibodies were expressed in *E. coli* BL21 (DE3)-Gold strain (Agilent Technologies) competent cells. Cells were plated and starter cultures inoculated from single colonies were grown overnight for 15 h at 37 °C at 200 rpm in LB medium supplemented with kanamycin (50 µg mL^−1^). Glycerol stocks were made by taking 700 µL of the starter cells supplemented with 700 µL glycerol, and stored at −80 °C. Growth was started in 1 L LB medium supplemented with kanamycin (50 µg mL^−1^) at 30 °C for about 7 h (to OD_600_ = 0.6–0.8), IPTG was added to a final concentration of 1 mM. After overnight expression at 30 °C, cells were harvested by centrifugation at 7500 rpm (JA-8.1 rotor, Beckmann Coulter) and resuspended in 45 mL 20 mM sodium phosphate (pH 8.0) buffer supplemented with 10 mM imidazole and one tablet of Roche Complete EDTA-free protease inhibitor cocktail. Cells were then lysed using sonication for 5 min at 40% amplitude in 15 s on and 45 s off cycles. The supernatant containing the protein was separated from cell debris using centrifugation at 18 000 rpm (JA-20 rotor, Beckmann Coulter). The cleared lysate was loaded onto a Ni^2+^-NTA Superflow column (Qiagen), equilibrated with 20 mM sodium phosphate buffer (pH 8.0) containing 10 mM imidazole. Non-specifically bound proteins were then washed away with 100 mL of 20 mM NaP buffer containing 60 mM imidazole, and the his-tagged DesAb was eluted with 20 mM NaP buffer containing 200 mM imidazole. The purity of the collected fractions was assessed by SDS-PAGE, and only the purest fractions were collected and was then subjected to gel filtration using a HiLoad 16/600 Superdex 75 pg column on an Äkta Pure system (GE Healthcare), and the peak corresponding to the monomeric DesAb was collected in 2 mL tubes (Corning) on ice. The column was equilibrated with 20 mM NaP 100 mM NaCl buffer at pH 8. Finally, the collected concentrated to a concentration between 10 µM and 30 µM *via* an Amicon Ultra – 15/10k *M*_w_. cut-off filter (Millipore), flash frozen and stored in the −80 °C.

### IAPP/biotin-IAPP purification

IAPP peptides were purchased from AlexoTech (Sweden, article no.: AI-452-10) and biotin-IAPP peptides were purchased from AnaSpec (USA, cat. number: AS-64451-05). Before running kinetics and binding affinity experiments, the purchased IAPP/biotin-IAPP peptide was dissolved in 6 M GuHCl (pH 8) and incubated for at least 1 h on ice. This solution was then subjected to gel filtration using a HiPrep 16/60 Sephacryl S-100 HR column on an Äkta Pure system (GE Healthcare), and the peak corresponding to the monomeric IAPP/biotin-IAPP was collected in low-binding test tubes (Corning) on ice. The column was equilibrated with 35 mM sodium acetate buffer supplemented with 150 mM KCl at pH 5.3.

### Aggregation assay for IAPP

Solutions were prepared containing monomeric 3 µM IAPP peptide in the presence of increasing amounts of each DesAb/monobody (90% v/v of 3.33 µM IAPP peptide and 10% v/v of DesAbs/monobodies, final buffer would be 31.5 mM sodium acetate 135 mM KCl 10 mM NaCl 2 mM NaP, supplemented with 20 µM ThT). Seeded experiments were performed in the presence of 40% preformed fibrils that were prepared freshly the same day with increasing antibody-to-IAPP monomer ratio (0 : 1, 1 : 8, 1 : 4 and 1 : 2). Each sample was then pipetted into multiple wells of a low-binding 96-well half-area plate of black polystyrene with a clear bottom and polyethylene glycol coating (Corning 3881) (80 µl per well). Plates were sealed to prevent evaporation. Aggregation assays were performed at 37 °C under quiescent conditions using a CLARIO or OMEGA star plate reader (BMG Labtech). The ThT fluorescence was measured through the bottom of the plate every minute with an excitation filter of 440 nm and an emission filter of 480 nm.

### BLI binding affinity measurements for IAPP

BLI measurements were performed using an Octet-BLI K2 system (ForteBio). All assays were carried out in a black 96-well plate, 200 µl per well, and all sensors were subjected to prehydration in the assay buffer for at least 15 min before usage. The assay plate was kept at 25 °C throughout the entire experiment. Anti-monomeric IAPP design binding assays were carried out in a buffer containing 35 mM sodium acetate, 150 mM KCl (pH 5.3) and 0.05% Tween20, shake speed 300. First, two Octet® SAX Biosensors (sample and reference) were preincubated in buffer for 15 min. Assay program consisted of a 180 s baseline in buffer; around 400 s (around 0.8 nm signal increase) loading using 1 µM purified monomeric biotin-IAPP; 180 s wash in buffer; 200 s baseline in buffer; 240 s association in different concentrations of anti-IAPP DesAb/monobody for the sample sensor and buffer for the reference sensor; and 240 s dissociation in buffer. 10 mM Glycine pH 2.0 was used for regeneration. Anti-fibrillar IAPP design binding assays were carried out in a buffer containing 35 mM sodium acetate, 150 mM KCl (pH 5.3) and 0.05% Tween20, shake speed 300. First, two Octet® AR2G Biosensors (sample and reference) were preincubated in buffer for 15 min. Assay program consisted of a 180 s baseline in buffer; 300 s activation in 20 mM EDC 10 mM NHS buffer; 1200 s (around 0.8 nm signal increase) loading using 1 µM purified monomeric biotin-IAPP; 180 s wash in buffer; 200 s baseline in buffer; 240 s association in different concentrations of anti-IAPP DesAb/monobody for the sample sensor and buffer for the reference sensor; and 240 s dissociation in buffer. 10 mM Glycine pH 2.0 was used for regeneration.

### Fluorescence microscopy

#### Glass coverslip passivation

The coating was based on previous reports.^60–62^ Briefly, glass coverslips were first cleaned with an argon plasma cleaner (PDC-002, Harrick Plasma) for 10 min. Following this, a polydimethylsiloxane (PDMS) gasket (Sigma, GBL103250-10 EA) was attached to the surface. 6 µL of coating solution (a mixture of Rain-X and isopropanol using 1 : 1 ratio and filtered using a 200 nm filter) was loaded into each well. Critically, the coating solution must be passed through a filter (Millex, SLGV004SL) prior to use. The coating buffer was left to dry naturally. The coated coverslip can be stored at room temperature for 2 weeks. The coating can last up to a few months, but we recommend use it within 2 weeks to prevent bacterial contamination. Rain-X (Rain-X Rain Repellent 200 mL) used in work was purchased from a local shop (Halfords, Cambridge, UK, CB5 8WR).

#### Small IAPP aggregates preparation

SEC-purified 5 µM monomeric IAPP peptide in PBS buffer was used for IAPP aggregates preparation. After 4 h incubation in a low-binding 96-well half-area plate of black polystyrene with a clear bottom and polyethylene glycol coating (Corning 3881) at 37 °C under quiescent conditions, 10 mg mL^−1^ HSA (final concentration after mixed with IAPP aggregates) was added to prevent the growth of the aggregates^52^ and the samples were taken out of the wells and used for imaging.

#### IAPP detection and single-molecule pull-down (SiMPull) experiment

The coated coverslip needs to be rinsed 2× with PBS by pipetting solution in and out of the wells, followed by incubation with NeutrAvidin solution with the desired concentration and incubation time. We used 0.1 mg mL^−1^ for 15 min as the default. Once NeutrAvidin incubation was complete, the wells were rinsed 3× with PBS by pipetting PBS in and out again. 1% F-127 solution (Invitrogen, P6866), made by mixing 10% stock with PBS and passing through a 200 nm filter, was loaded into the imaging wells on coverslip and incubated for 45 min. F-127 residues were then washed by rinsing the coverslip 3× using PBST (PBS + 0.05% Tween20). Where necessary, a BSA blocking step (1% BSA in PBST and incubated for 20 min, with 2× PBST washing at the end) can be performed this point. Then 10 nM of relevant biotinylated capture antibodies (10 µL) were diluted in the PBST and incubated in each well for 5 min. After incubation, coverslips were rinsed 3× with PBST. Samples should then be loaded onto wells. We incubated IAPP aggregates for 15 min and biofluids (serum) for 90 min.

For IAPP monomers detection, the biotinylated IAPP were directly loaded on the NeutrAvidin coated coverslip for 30 min at a concentration of 1 µM. Once the sample incubation was complete, coverslips require rinsing 3× with PBST and before incubation with detection antibodies. For IAPP aggregates detection, we incubated with detection antibodies at 500 pM for 5 min. For T2D serum, we incubated 5 nM detection antibody for 20 min. Following detection antibody incubation, coverslips were once again washed 3× with PBST. Before the imaging, wells were filled with PBS. AF647 labelled commercial anti-IAPP antibody E5 (amylin (E-5): sc-377530) was purchased from Insight Biotechnology (cat: sc-377530-AF647). The target epitope of *e*% is within amino acids 40–89 at the C-terminus of pre-pro-IAPP, corresponding to amino acids 7–37 at the C-terminus of IAPP.

#### Super-resolution fluorescence microscopy

We performed direct stochastic optical reconstruction microscopy (dSTORM) to visualise labelled IAPP aggregates with super-resolution. Briefly, the dSTORM buffer (glucose oxidase (2 mg mL^−1^, Sigma, G7141-250KU), catalase (52 µg mL^−1^, Sigma, C3515) and cysteamine (7 mg mL^−1^, Sigma, M9768-5G) at pH 8.0) was added to the imaging wells containing the fluorescently-labelled aggregates. The imaging well was carefully sealed with a coverslip to minimise oxygen penetration. The data acquitision was performed using a 638 nm laser (Cobolt MLD 638, cobalt) at 180 mW with a constant 405 nm pulse (LBX-405-50-CIR-PP, Oxxius). 5000 frames were recorded with an exposure time of 30 ms.

#### Data analysis

Data analysing was performed using codes reported in our previous works.^63^ Briefly, single-particle counting was performed using a single-particle localisation engine in ThunderSTORM.^64^ The images were processed using a wavelet filter and the localisations were identified using a hybrid threshold of 1.5 × std (Wave.F1). The super-resolution imaging reconstructions were done *via* drift correction,^65^ peak fitting and post-fit analysis. The morphological analysis module in our code was not used in this work.^66^ The code used for this work is available at https://github.com/YPZ858/DF-single-molecule-counting (single-molecule counting) and https://github.com/YPZ858/Super-res-code/issues (super-resolution imaging).^63^

## Author contributions

J. L., P. S., D. K. and M. V. designed research; J. L., Y. P. Z., S. C. and P. S. performed research; all authors analyzed data and wrote the paper.

## Conflicts of interest

There are no conflicts to declare.

## Supplementary Material

SC-017-D5SC01427A-s001

## Data Availability

All study data are included in the article and supplementary information (SI). Supplementary information: patient metadata, sequences of all designed antibody-like scaffolds, and biochemical characterisation of DesAbs and monobodies. It also provides TIRF/SiMPull single-molecule imaging data for monomeric and aggregated IAPP, buffer controls, and type 2 diabetes serum. See DOI: https://doi.org/10.1039/d5sc01427a.

## References

[cit1] Sun H., Saeedi P., Karuranga S., Pinkepank M., Ogurtsova K., Duncan B. B., Stein C., Basit A., Chan J. C., Mbanya J. C. (2022). IDF diabetes atlas: Global, regional and country-level diabetes prevalence estimates for 2021 and projections for 2045. Diabetes Res. Clin. Pract..

[cit2] Saeedi P., Salpea P., Karuranga S., Petersohn I., Malanda B., Gregg E. W., Unwin N., Wild S. H., Williams R. (2020). Mortality attributable to diabetes in 20–79 years old adults, 2019 estimates: Results from the international diabetes federation diabetes atlas. Diabetes Res. Clin. Pract..

[cit3] Chen L., Magliano D. J., Zimmet P. Z. (2012). The worldwide epidemiology of type 2 diabetes mellitus—present and future perspectives. Nat. Rev. Endocrinol..

[cit4] Williams R., Karuranga S., Malanda B., Saeedi P., Basit A., Besançon S., Bommer C., Esteghamati A., Ogurtsova K., Zhang P. (2020). Global and regional estimates and projections of diabetes-related health expenditure: Results from the international diabetes federation diabetes atlas. Diabetes Res. Clin. Pract..

[cit5] Ahmad E., Lim S., Lamptey R., Webb D. R., Davies M. J. (2022). Type 2 diabetes. Lancet.

[cit6] Zheng Y., Ley S. H., Hu F. B. (2018). Global aetiology and epidemiology of type 2 diabetes mellitus and its complications. Nat. Rev. Endocrinol..

[cit7] DeFronzo R. A., Ferrannini E., Groop L., Henry R. R., Herman W. H., Holst J. J., Hu F. B., Kahn C. R., Raz I., Shulman G. I. (2015). Type 2 diabetes mellitus. Nat. Rev. Dis. Primers.

[cit8] Westermark P., Andersson A., Westermark G. T. (2011). Islet amyloid polypeptide, islet amyloid, and diabetes mellitus. Physiol. Rev..

[cit9] Kahn S. E., D'Alessio D. A., Schwartz M. W., Fujimoto W. Y., Ensinck J. W., Taborsky Jr G. J., Porte Jr D. (1990). Evidence of cosecretion of islet amyloid polypeptide and insulin by β-cells. Diabetes.

[cit10] Westermark P., Engström U., Johnson K. H., Westermark G. T., Betsholtz C. (1990). Islet amyloid polypeptide: Pinpointing amino acid residues linked to amyloid fibril formation. Proc. Natl. Acad. Sci. U. S. A..

[cit11] Westermark G. T., Westermark P., Berne C., Korsgren O. (2008). Widespread amyloid deposition in transplanted human pancreatic islets. N. Engl. J. Med..

[cit12] Abedini A., Plesner A., Cao P., Ridgway Z., Zhang J., Tu L.-H., Middleton C. T., Chao B., Sartori D. J., Meng F. (2016). Time-resolved studies define the nature of toxic iapp intermediates, providing insight for anti-amyloidosis therapeutics. eLife.

[cit13] Caillon L., Hoffmann A. R., Botz A., Khemtemourian L. (2016). Molecular structure, membrane interactions, and toxicity of the islet amyloid polypeptide in type 2 diabetes mellitus. J. Diabetes Res..

[cit14] Haataja L., Gurlo T., Huang C. J., Butler P. C. (2008). Islet amyloid in type 2 diabetes, and the toxic oligomer hypothesis. Endocr. Rev..

[cit15] Milardi D., Gazit E., Radford S. E., Xu Y., Gallardo R. U., Caflisch A., Westermark G. T., Westermark P., Rosa C. L., Ramamoorthy A. (2021). Proteostasis of islet amyloid polypeptide: A molecular perspective of risk factors and protective strategies for type ii diabetes. Chem. Rev..

[cit16] Rehn F., Kraemer-Schulien V., Bujnicki T., Bannach O., Tschoepe D., Stratmann B., Willbold D. (2024). Iapp-oligomerisation levels in plasma of people with type 2 diabetes. Sci. Rep..

[cit17] Wirth F., Heitz F. D., Seeger C., Combaluzier I., Breu K., Denroche H. C., Thevenet J., Osto M., Arosio P., Kerr-Conte J. (2023). A human antibody against pathologic iapp aggregates protects beta cells in type 2 diabetes models. Nat. Commun..

[cit18] Kulenkampff K., Wolf Perez A.-M., Sormanni P., Habchi J., Vendruscolo M. (2021). Quantifying misfolded protein oligomers as drug targets and biomarkers in Alzheimer and parkinson diseases. Nat. Rev. Chem..

[cit19] De Genst E., Messer A., Dobson C. M. (2014). Antibodies and protein misfolding: From structural research tools to therapeutic strategies. Biochim. Biophys. Acta, Proteins Proteomics.

[cit20] Sormanni P., Aprile F. A., Vendruscolo M. (2018). Third generation antibody discovery methods: *In silico* rational design. Chem. Soc. Rev..

[cit21] Perchiacca J. M., Ladiwala A. R. A., Bhattacharya M., Tessier P. M. (2012). Structure-based design of conformation-and sequence-specific antibodies against amyloid β. Proc. Natl. Acad. Sci. U. S. A..

[cit22] Aprile F. A., Sormanni P., Podpolny M., Chhangur S., Needham L.-M., Ruggeri F. S., Perni M., Limbocker R., Heller G. T., Sneideris T. (2020). Rational design of a conformation-specific antibody for the quantification of Aβ oligomers. Proc. Natl. Acad. Sci. U. S. A..

[cit23] Sormanni P., Aprile F. A., Vendruscolo M. (2015). Rational design of antibodies targeting specific epitopes within intrinsically disordered proteins. Proc. Natl. Acad. Sci. U. S. A..

[cit24] Wolf Pérez A.-M., Sormanni P., Andersen J. S., Sakhnini L. I., Rodriguez-Leon I., Bjelke J. R., Gajhede A. J., De Maria L., Otzen D. E., Vendruscolo M. (2019). In vitro and in silico assessment of the developability of a designed monoclonal antibody library. MAbs.

[cit25] Fischman S., Ofran Y. (2018). Computational design of antibodies. Curr. Opin. Struct. Biol..

[cit26] Baran D., Pszolla M. G., Lapidoth G. D., Norn C., Dym O., Unger T., Albeck S., Tyka M. D., Fleishman S. J. (2017). Principles for computational design of binding antibodies. Proc. Natl. Acad. Sci. U. S. A..

[cit27] Aprile F. A., Sormanni P., Perni M., Arosio P., Linse S., Knowles T. P., Dobson C. M., Vendruscolo M. (2017). Selective targeting of primary and secondary nucleation pathways in Aβ_42_ aggregation using a rational antibody scanning method. Sci. Adv..

[cit28] Lin J., Figazzolo C., Metrick M. A., Sormanni P., Vendruscolo M. (2021). Computational maturation of a single-domain antibody against Aβ_42_ aggregation. Chem. Sci..

[cit29] Kulenkampff K., Emin D., Staats R., Zhang Y. P., Sakhnini L., Kouli A., Rimon O., Lobanova E., Williams-Gray C. H., Aprile F. A. (2022). An antibody scanning method for the detection
of α-synuclein oligomers in the serum of Parkinson's disease patients. Chem. Sci..

[cit30] Muyldermans S. (2013). Nanobodies: Natural single-domain antibodies. Annu. Rev. Biochem..

[cit31] Harmsen M. M., De Haard H. J. (2007). Properties, production, and applications of camelid single-domain antibody fragments. Appl. Microbiol. Biotechnol..

[cit32] Kondo T., Iwatani Y., Matsuoka K., Fujino T., Umemoto S., Yokomaku Y., Ishizaki K., Kito S., Sezaki T., Hayashi G. (2020). Antibody-like proteins that capture and neutralize SARS-CoV-2. Sci. Adv..

[cit33] Koide A., Bailey C. W., Huang X., Koide S. (1998). The fibronectin type iii domain as a scaffold for novel binding proteins. J. Mol. Biol..

[cit34] Axelrod D. (2001). Total internal reflection fluorescence microscopy in cell biology. Traffic.

[cit35] Heilemann M., Van De Linde S., Schüttpelz M., Kasper R., Seefeldt B., Mukherjee A., Tinnefeld P., Sauer M. (2008). Subdiffraction-resolution fluorescence imaging with conventional fluorescent probes. Angew. Chem., Int. Ed..

[cit36] Bhuskute K. R., Kikuchi K., Luo Z., Kaur A. (2024). Visualizing amyloid assembly at the nanoscale: Insights from super-resolution imaging. Analysis Sensing.

[cit37] López-Mirabal H. R., Winther J. R. (2008). Redox characteristics of the eukaryotic cytosol. Biochim. Biophys. Acta, Mol. Cell Res..

[cit38] Bradbury A. R., Sidhu S., Dübel S., McCafferty J. (2011). Beyond natural antibodies: The power of *in vitro* display technologies. Nat. Biotechnol..

[cit39] Morrison M. S., Wang T., Raguram A., Hemez C., Liu D. R. (2021). Disulfide-compatible phage-assisted continuous evolution in the periplasmic space. Nat. Commun..

[cit40] Müller A., Hoffmann J. H., Meyer H. E., Narberhaus F., Jakob U., Leichert L. I. (2013). Nonnative disulfide bond formation activates the σ32-dependent heat shock response in escherichia coli. J. Bacteriol..

[cit41] Liu H., Schittny V., Nash M. A. (2019). Removal of a conserved disulfide bond does not compromise mechanical stability of a vhh antibody complex. Nano Lett..

[cit42] Christ D. (2017). Faster, deeper, smaller—the rise of antibody-like scaffolds. Nat. Biotechnol..

[cit43] Gebauer M., Skerra A. (2020). Engineered protein scaffolds as next-generation therapeutics. Annu. Rev. Pharmacol. Toxicol..

[cit44] Hoyt E. A., Cal P. M., Oliveira B. L., Bernardes G. J. (2019). Contemporary approaches to site-selective protein modification. Nat. Rev. Chem..

[cit45] Chalker J. M., Bernardes G. J., Lin Y. A., Davis B. G. (2009). Chemical modification of proteins at cysteine: Opportunities in chemistry and biology. Chem.–Asian J..

[cit46] Seo M. J., Jeong K. J., Leysath C. E., Ellington A. D., Iverson B. L., Georgiou G. (2009). Engineering antibody fragments to fold in the absence of disulfide bonds. Protein Sci..

[cit47] Woërn A., Pluëckthun A. (1998). An intrinsically stable antibody scfv fragment can tolerate the loss of both disulfide bonds and fold correctly. FEBS Lett..

[cit48] Li Z., Krippendorff B.-F., Shah D. K. (2017). Influence of molecular size on the clearance of antibody fragments. Pharm. Res..

[cit49] Sormanni P., Aprile F. A., Vendruscolo M. (2015). The CamSol method of rational design of protein mutants with enhanced solubility. J. Mol. Biol..

[cit50] Elenbaas B. O., Khemtemourian L., Killian J. A., Sinnige T. (2022). Membrane-catalyzed aggregation of islet amyloid polypeptide is dominated by secondary nucleation. Biochemistry.

[cit51] Rodriguez Camargo D. C., Chia S., Menzies J., Mannini B., Meisl G., Lundqvist M., Pohl C., Bernfur K., Lattanzi V., Habchi J. (2021). Surface-catalyzed secondary nucleation dominates the generation of toxic iapp aggregates. Front. Mol. Biosci..

[cit52] Bellomo G., Bologna S., Cerofolini L., Paciotti S., Gatticchi L., Ravera E., Parnetti L., Fragai M., Luchinat C. (2019). Dissecting the interactions between human serum albumin and α-synuclein: New insights on the factors influencing α-synuclein aggregation in biological fluids. J. Phys. Chem. B.

[cit53] Reyes Barcelo A. A., Gonzalez-Velasquez F. J., Moss M. A. (2009). Soluble aggregates of the amyloid-β peptide are trapped by serum albumin to enhance amyloid-β activation of endothelial cells. J. Biol. Eng..

[cit54] Arndt J. W., Qian F., Smith B. A., Quan C., Kilambi K. P., Bush M. W., Walz T., Pepinsky R. B., Bussière T., Hamann S. (2018). Structural and kinetic basis for the selectivity of aducanumab for aggregated forms of amyloid-β. Sci. Rep..

[cit55] Cohen S. I., Linse S., Luheshi L. M., Hellstrand E., White D. A., Rajah L., Otzen D. E., Vendruscolo M., Dobson C. M., Knowles T. P. (2013). Proliferation of amyloid-β42 aggregates occurs through a secondary nucleation mechanism. Proc. Natl. Acad. Sci. U. S. A..

[cit56] Buell A. K., Galvagnion C., Gaspar R., Sparr E., Vendruscolo M., Knowles T. P., Linse S., Dobson C. M. (2014). Solution conditions determine the relative importance of nucleation and growth processes in α-synuclein aggregation. Proc. Natl. Acad. Sci. U. S. A..

[cit57] Habchi J., Chia S., Limbocker R., Mannini B., Ahn M., Perni M., Hansson O., Arosio P., Kumita J. R., Challa P. K., Cohen S. I., Linse S., Dobson C. M., Knowles T. P., Vendruscolo M. (2017). Systematic development of small molecules to inhibit specific microscopic steps of Aβ_42_ aggregation in Alzheimer's disease. Proc. Natl. Acad. Sci. U. S. A..

[cit58] Metrick M. A., do Carmo Ferreira N., Saijo E., Hughson A. G., Kraus A., Orrú C., Miller M. W., Zanusso G., Ghetti B., Vendruscolo M. (2019). Million-fold sensitivity enhancement in proteopathic seed amplification assays for biospecimens by hofmeister ion comparisons. Proc. Natl. Acad. Sci. U. S. A..

[cit59] Galvagnion C., Buell A. K., Meisl G., Michaels T. C., Vendruscolo M., Knowles T. P., Dobson C. M. (2015). Lipid vesicles trigger α-synuclein aggregation by stimulating primary nucleation. Nat. Chem. Biol..

[cit60] Furlepa M., Zhang Y. P., Lobanova E., Kahanawita L., Vivacqua G., Williams-Gray C. H., Klenerman D. (2024). Single-molecule characterization of salivary protein aggregates from Parkinson's disease patients: A pilot study. Brain Commun..

[cit61] Zhang Y. P., Lobanova E., Dworkin A., Furlepa M., Yang W. S., Burke M., Meng J. X., Potter N., Sala R. L., Kahanawita L. (2024). Improved imaging surface for quantitative single-molecule microscopy. ACS Appl. Mater. Interfaces.

[cit62] Lobanova E., Zhang Y. P., Emin D., Brelstaff J., Kahanawita L., Malpetti M., Quaegebeur A., Triantafilou K., Triantafilou M., Zetterberg H. (2024). Asc specks as a single-molecule fluid biomarker of inflammation in neurodegenerative diseases. Nat. Commun..

[cit63] Zhang Y. P., Lobanova E., Emin D., Lobanov S. V., Kouli A., Williams-Gray C. H., Klenerman D. (2023). Imaging protein aggregates in Parkinson's disease serum using aptamer-assisted single-molecule pull-down. Anal. Chem..

[cit64] Ovesný M., Křížek P., Borkovec J., Švindrych Z., Hagen G. M. (2014). Thunderstorm: A comprehensive ImageJ plug-in for palm and storm data analysis and super-resolution imaging. Bioinformatics.

[cit65] Fazekas F. J., Shaw T. R., Kim S., Bogucki R. A., Veatch S. L. (2021). A mean shift algorithm for drift correction in localization microscopy. Biophys. Rep..

[cit66] Legland D., Arganda-Carreras I., Andrey P. (2016). Morpholibj: Integrated library and plugins for mathematical morphology with ImageJ. Bioinformatics.

