# Supplementary material for: Single-molecule imaging of small aggregates of IAPP in type 2 diabetes serum with rationally-designed antibody-like scaffolds
Source: Chem Sci. 2025 Dec 11;17(5):2748–66. doi: 10.1039/d5sc01427a (PMC12696916; doi:10.1039/d5sc01427a)
Supplement: SC-017-D5SC01427A-s001 [file SC-017-D5SC01427A-s001.pdf]

Supplementary Information (SI) for Chemical Science.
This journal is © The Royal Society of Chemistry 2025

SUPPORTING INFORMATION

Single-molecule imaging of small aggregates of IAPP in type 2 diabetes serum with rationally-designed antibody-like scaffolds

Jiacheng Lin[1,2,‡], Yu P. Zhang[2,‡], Sean Chia, David Klenerman[2,*],
Pietro Sormanni[1,2,*] and Michele Vendruscolo[1,2,*]

[1]*Centre for Misfolding Diseases, University of Cambridge, Cambridge CB2 1EW, UK*
[2]*Yusuf Hamied Department of Chemistry, University of Cambridge, Cambridge CB2 1EW, UK*

Table S1. Patient information of T2D serum used in this work.

Patient: Patient ID	Patient: Ethnicity	Patient Gender	Age	Serial Number
900483669	Unknown	Male	60 Years	W000144165
900483674	Unknown	Male	66 Years	W000144176
900495991	Unknown	Male	65 Years	W000158215
900458272	Unknown	Female	18 Years	W000119824
900470657	Unknown	Male	11 Years	W000131501
900470659	Hispanic/Latino	Female	19 Years	W000131622

Table S2. Summary of biochemical and diagnostic properties of antibody constructs used in this work.

Construct	Aggregation Inhibition#	Monomer KD	(Fibril KD	TIRF/SiMPull Imaging*	Serum Detectability	Diagnostic Value
**DesAb$_{9-17}$**	Delays IAPP aggregation (Strong)	High nM to low µM (2.07 µM)	ND	Monomers (low signal); Aggregates (low signal)	No significant serum signal	Strong in vitro binder; a high background in serum limits direct diagnostic use.
**DesAb$_{12-18}$**	Delays IAPP aggregation (Strong)	No stable monomer signal)	ND	Not tested in TIRF panels	Not tested	Likely oligomer-specific; it could probe transient species rather than bulk diagnostic marker.
**DesAb$_{15-22}$**	Delays IAPP aggregation (Moderate)	High nM to low µM (0.50 µM)	Low µM	Not tested in TIRF panels	Not tested	Moderate binder to both monomer and fibril; potential tool for mechanistic studies, less for diagnostics.
**DesAb$_{19-26}$**	Delays IAPP aggregation (Strong)	High nM to low µM (0.40 µM)	ND	Monomers (Moderate signal); Aggregates (Strong signal); super-resolves size/shape differences	Significant serum detection	Best diagnostic candidate: detects both soluble and serum aggregates with morphological resolution.
**DesAb$_{20-28}$**	Delays IAPP aggregation (Strong)	High nM to low µM (0.20 µM)	ND	Monomers (Strong signal); Aggregates (Strong signal); super-resolves size difference	Significant serum detection	Excellent probe of both in vitro aggregates and serum, with super-resolution capabilities.
**DesAb$_{22-29}$**	No inhibition of IAPP aggregation despite binding	Low µM (binds monomer,0.53 µM)	Low µM	Monomers (Strong signal); Aggregates (no difference from blank);	ND	Bystander binder; binds but does not perturb aggregation; less useful diagnostically.
**DesAb$_{26-32}$**	Delays IAPP aggregation (Strong)	High nM to low µM (1.4 µM)	ND	Monomers (Moderate signal);	ND	Good aggregation inhibitor, but

				Aggregates (no difference from blank)		low imaging signal suggests limited diagnostic utility.
**DesAb**$_{26-34}$	Delays IAPP aggregation (Strong)	High nM to low µM (0.32 µM)	low µM	Monomers (Strong signal); Aggregates (no difference from blank)	ND	Strong monomer binder, but no serum signal; better for in vitro assays than direct diagnostics.
**DesAb**$_{30-37}$	Delays IAPP aggregation (Weak)	High nM to low µM (0.41 µM)	low µM	Not tested in TIRF panels	Not tested	Weak aggregation inhibitor
**DesAb**$_{neg}$	No inhibition of IAPP aggregation	ND	ND	Negative control, some non-specific binding to monomers	Some non-specific binding	Benchmark negative control.
**Monobody**$_{9-17}$	Delays IAPP aggregation (Weak)	ND	ND	Monomers (weak signal); Aggregates (weak signal)	Significant serum detection	Compact binder with serum signal; promising scaffold for diagnostics after affinity maturation.
**Monobody**$_{19-26}$	Delays IAPP aggregation (Moderate)	ND	ND	Monomers (weak signal); Aggregates (weak signal, no difference from blank) X	ND	Good in vitro imaging probe but lacks serum sensitivity as is.
**Monobody**$_{20-28}$	Delays IAPP aggregation (weak)	ND	ND	Monomers (weak signal); Aggregates no difference from blank)	ND	Moderate in vitro activity; needs further optimization for diagnostics.
**Monobody**$_{26-34}$	Delays IAPP aggregation (weak)	ND	ND	Monomers (weak signal); Aggregates (no difference from blank)	ND	Lowest affinity scaffold; mainly useful as negative control for monobody series.
**Monobody**$_{neg}$	No inhibition of IAPP aggregation	ND	ND	Negative control	Negative	Benchmark negative control.

The underlined DesAbs were selected for monobody development based on their promising performance.

At a DeSab:IAPP ratio of 1:2. The inhibition level is quantified by comparing the time taken for the

reference and DeSab incubated conditions to reach a relative fibril mass of 50%. "Strong" refers to a ~100% delay, "moderate" to ~50% delay, and "weak" to ~15% delay.
* Recombinant proteins were used for imaging.

Table S3. Degree of Labelling (DOL) and non-specific absorption on different surfaces

Name	DOL	Absorption on NA surface	Absorption on E5 surface
**DesAb$_{neg}$**	1.42	6.60	33.42
**DesAb$_{9\text{-}17}$**	1.37	5.79	20.29
**DesAb$_{19\text{-}26}$**	2.86	38.35	36.17
**DesAb$_{20\text{-}28}$**	1.40	25.65	68.23
**DesAb$_{22\text{-}29}$**	1.35	45.75	30.44
**DesAb$_{26\text{-}32}$**	1.53	34.08	38.19
**DesAb$_{26\text{-}34}$**	1.12	184.29	35.38
**Monobody$_{neg}$**	0.94	7.67	35.54
**Monobody$_{9\text{-}17}$**	0.79	8.63	21.58
**Monobody$_{19\text{-}26}$**	0.87	5.06	46.27
**Monobody$_{20\text{-}28}$**	0.77	2.90	49.50
**Monobody$_{26\text{-}34}$**	0.75	13.48	27.17

M E V Q L V E S G G G L V Q P G G S L R L S **C** A A S G F N I 30

K D T Y I G W V R R A P G K G E E W V A S I Y P T N G Y T R 60

Y A D S V K G R F T I S A D T S K N T A Y L Q M N S L R A E 90

D T A V Y Y **C** A A G S **F E T L T L R** E E E A A A W G Q G T L 120

V T V S S G S H H H H H H

Scheme S1. Sequence of the sdAb scaffold used for the removal of the conserved disulfide bond. The sequence of the designed CDR3, along with the two cysteines for substitution, is highlighted in bold.

M V S S V P T K L E V V A A T P T S L L I S W D A **F E T L T**

L R V D Y Y R I T Y G E T G G N S P V Q E F T V P G S K S T

A T I S G L K P G V D Y T I T V Y A **V T G R G D S P A S S** S

P I S I N Y R T H H H H H H

Scheme S2. Sequence of the monobody construct FETLTLR(BC)-wt(FG).

M V S S V P T K L E V V A A T P T S L L I S W D A **G S F E T**

L T L R E E E V D Y Y R I T Y G E T G G N S P V Q E F T V P

G S K S T_A T I S G L K P G V D Y T I T V Y A **V T G R G D S**

P A S S S P I S I N Y R T H H H H H H

Scheme S3. Sequence of the monobody construct GSFETLTLREEE(BC)-wt(FG).

M V S S V P T K L E V V A A T P T S L L I S W D A **P A V T** V

D Y Y R I T Y G E T G G N S P V Q E F T V P G S K S T_A T I

S G L K P G V D Y T I T V Y A **F E T L T L R** S P I S I N Y R

T H H H H H H

Scheme S4. Sequence of the monobody construct wt(BC)-FETLTLR(FG).

M V S S V P T K L E V V A A T P T S L L I S W D A **P A V T** V

D Y Y R I T Y G E T G G N S P V Q E F T V P G S K S T_A T I

S G L K P G V D Y T I T V Y A **G S** **F E T L T L R E E E** S P I

S I N Y R T H H H H H H

Scheme S5. Sequence of the monobody construct wt(BC)-GSFETLTLREEE(FG).

M P T K L E V V A A T P T S L L I S W D A **F E T L T L R** V D

Y Y R I T Y G E T G G N S P V Q E F T V P G S K S T_A T I S

G L K P G V D Y T I T V Y A **V T G R G D S P A S S** S P I S I

N Y R T H H H H H H

Scheme S6. Sequence of the monobody construct N-4-FETLTLR(BC)-wt(FG).

M E V V A A T P T S L L I S W D A **F E T L T L R** V D Y Y R I

T Y G E T G G N S P V Q E F T V P G S K S T_A T I S G L K P

G V D Y T I T V Y A **A A A A S** S P I S I N Y R T H H H H H H

Scheme S7. Sequence of the monobody construct N-8-FETLTLR(BC)-AAAAS(FG).

M E V V A A T P T S L L I S W D A **G S F E T L T L R E E E** V

D Y Y R I T Y G E T G G N S P V Q E F T V P G S K S T_A T I

S G L K P G V D Y T I T V Y A **A A A A S** S P I S I N Y R T H

H H H H H

Scheme S8. Sequence of the monobody construct N-8-GSFETLTLREEE(BC)-AAAAS(FG).

M E V V A A T P T S L L I S W D A **F E T L T L R** V D Y Y R I

T Y G E T G G N S P V Q E F T V P G S K S T_A T I S G L K P

G V D Y T I T V Y A **E G Y Y S S Y** S P I S I N Y R T H H H H

H H

Scheme S9. Sequence of the monobody construct N-8-FETLTLR(BC)-EGYYSSY(FG).

M E V V A A T P T S L L I S W D A **F E T L T L R** V D Y Y R I

T Y G E T G G N S P V Q E F T V P G S K S T_A T I S G L K P

G V D Y T I T V Y A **P T S D Y G** S P I S I N Y R T H H H H H

H

Scheme S10. Sequence of the monobody construct N-8-FETLTLR(BC)-PTSDYG(FG).

M E V Q L V E S G G G L V Q P G G S L R L S A A A S G F N I 30

K D T Y I G W V R R A P G K G E E W V A S I Y P T N G Y T R 60

Y A D S V K G R F T I S A D T S K N T A Y L Q M N S L R A E 90

D T A V Y Y A A A G S **Designed CDR3** E E E A A A W G Q G T L 120

V T V S S G S H H H H H H

Scheme S11. Sequence of the conserved disulfide bond removed sdAb scaffold used for the scanning of IAPP. The sequences of the various designed CDR3 employed are in **Figure 3a**.

M E V V A A T P T S L L I S W D A **Designed binding loop** V D Y 30

Y R I T Y G E T G G N S P V Q E F T V P G S K S T A T I S G 60

L K P G V D Y T I T V Y A E G Y Y S S Y S S P I S I N Y R T 90

H H H H H H

Scheme S12. Sequence of the monobody scaffold used for the generation of a library of monobodies against IAPP. The sequences of the various designed binding loops employed are in **Figure 5a**.

M V S S V P T K L E V V A A T P T S L L I S W D A **P A V T**

V D Y Y R I T Y G E T G G N S P V Q E F T V P G S K S T A

T I S G L K P G V D Y T I T V Y A **V T G R G D S P A S S** S

P I S I N Y R T H H H H H H

Scheme S13. Sequence of the non-grafted monobody construct wt(BC)-wt(FG).

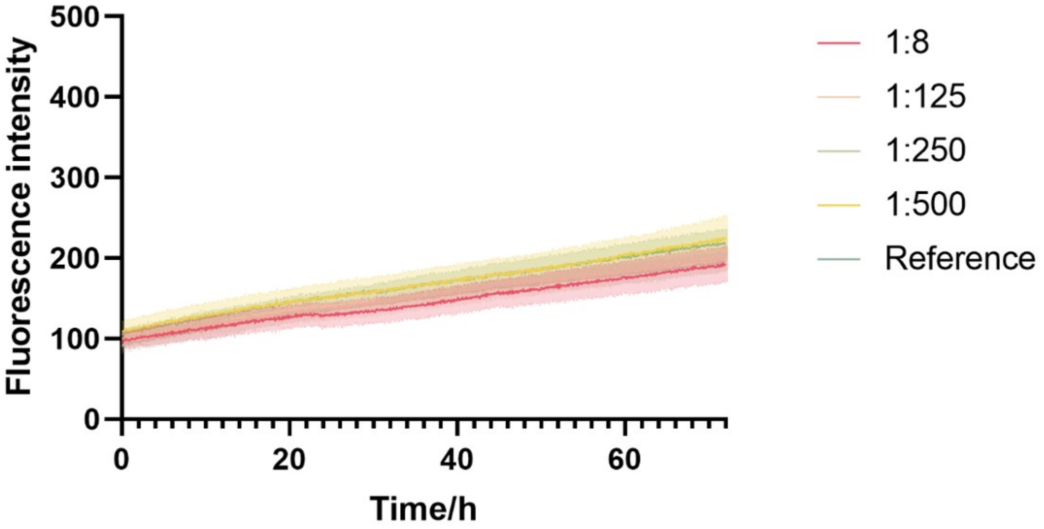

Figure S1. Aggregation assays of aS in the presence of FETLTLR_C23A_C97A. The aggregation process of aS was monitored at 40 μM concentration with 0.5% seeds of aS in the presence of increasing concentrations of the FETLTLR_C23A_C97A in triplicate at 37 °C under quiescent conditions. DesAb:aS ratios 0:1 (as reference, green), 1:500 (yellow), 1:250 (light green), 1:125 (light orange) and 1:8 (red). The seeds of aS were prepared at 200 μM, 42 °C, 3 days with stirring and sonicated before use.

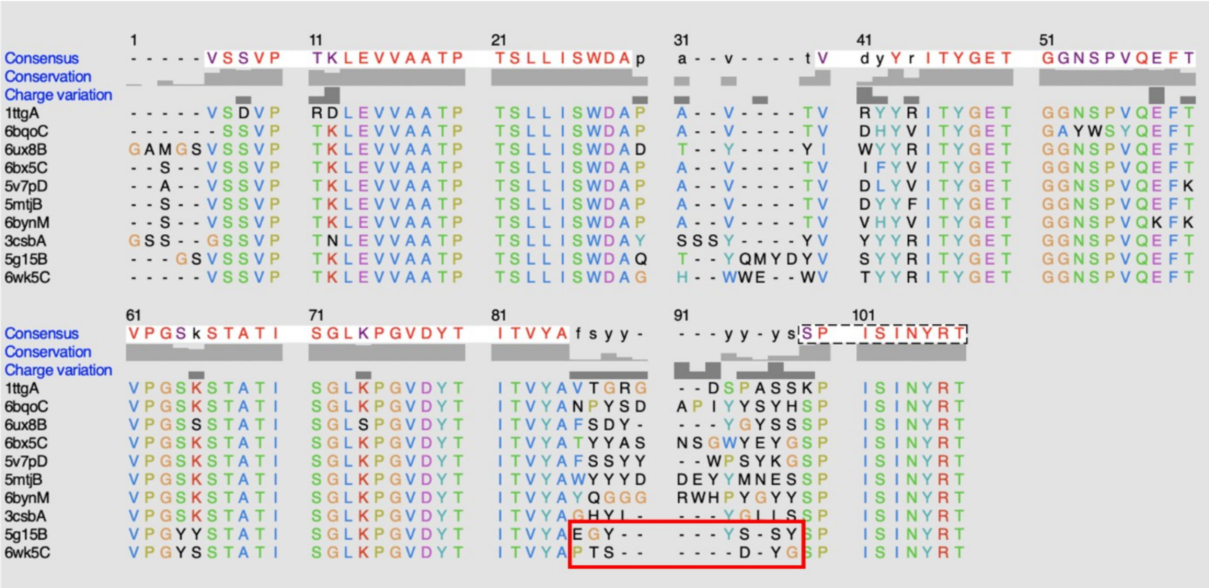

Figure S2. Multiple sequence alignment (MSA) of the monobody sequences with reported structures. The red box indicates the two short sequences used for the FG loop in this study.

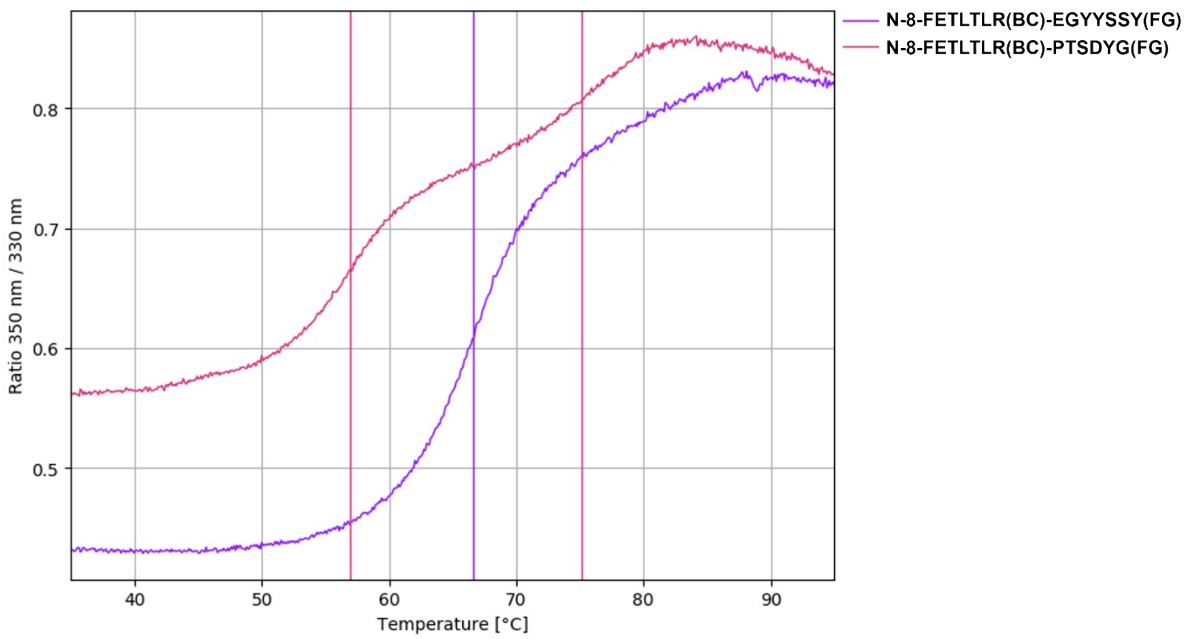

Figure S3. Thermal denaturation measurements. We characterised the two monobody constructs N-8-FETLTLR(BC)-EGYYSSY(FG) (purple) and N-8-FETLTLR(BC)-PTSDYG(FG) (red) using a Nanotemper Tycho NT.6 system.

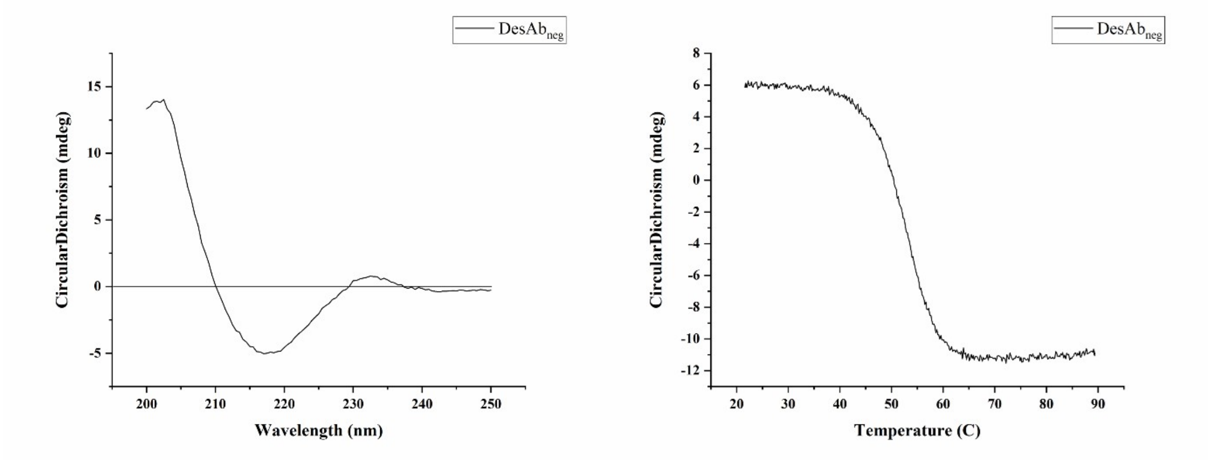

Figure S4. CD spectrum (left) and CD thermal denaturation spectrum (right) of the negative control DesAb$_{neg}$. The DesAb sample was measured at the concentration of 10 μM.

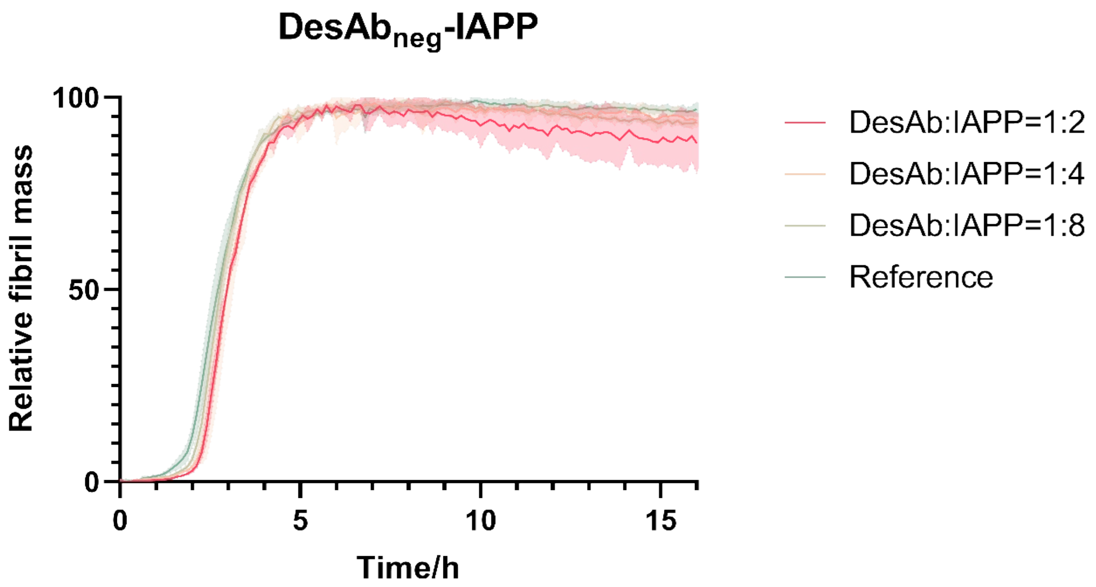

Figure S5. Aggregation assays of IAPP in the presence of the negative control DesAb. We monitored the aggregation process of IAPP at 3 μM concentration in the presence of increasing concentrations of the DesAb in quadruplicate at 37 °C under quiescent conditions. We tested the DesAb : IAPP ratios 0:1 (as reference, green), 1:8 (light green), 1:4 (orange) and 1:2 (red) finding in all cases a dose-depended delay of the aggregation process.

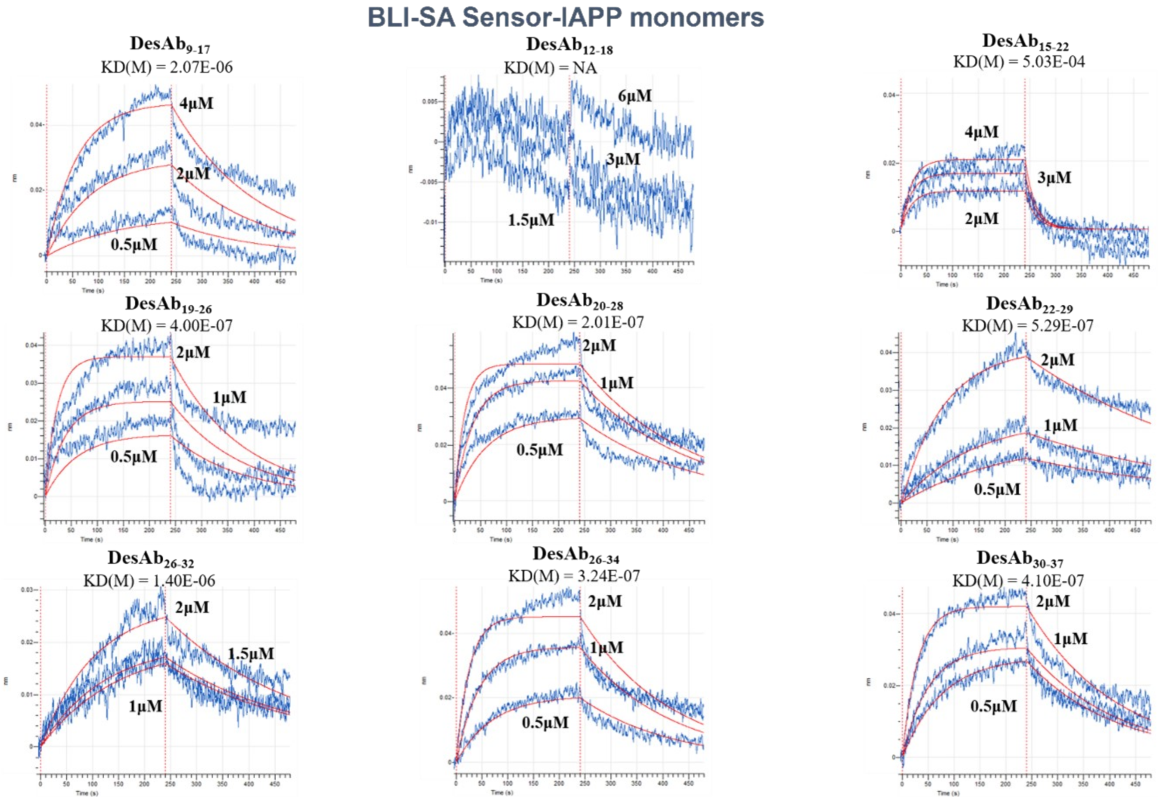

Figure S6. Measurement of the binding affinity of the DesAbs to monomeric IAPP. BLI binding traces (association and dissociation) obtained with SAX sensors loaded with monomeric biotin-IAPP. Association was monitored in wells containing different concentrations of DesAbs, as indicated in the figure.

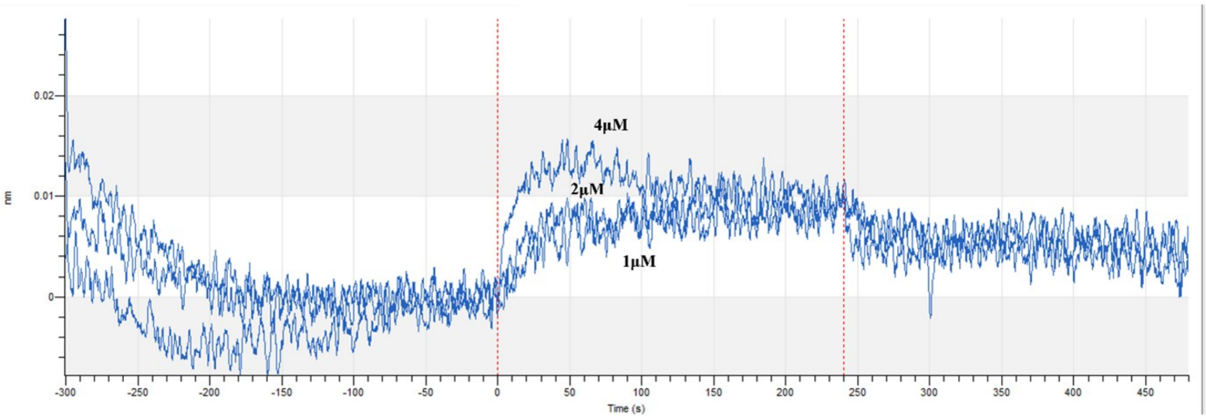

Figure S7. Measurement of the binding affinity of the negative control DesAb$_{neg}$ to monomeric IAPP. BLI binding traces (association and dissociation) obtained with SAX sensors loaded with monomeric biotin-IAPP. Association was monitored in wells containing different concentrations of DesAb$_{neg}$, as shown in the figure.

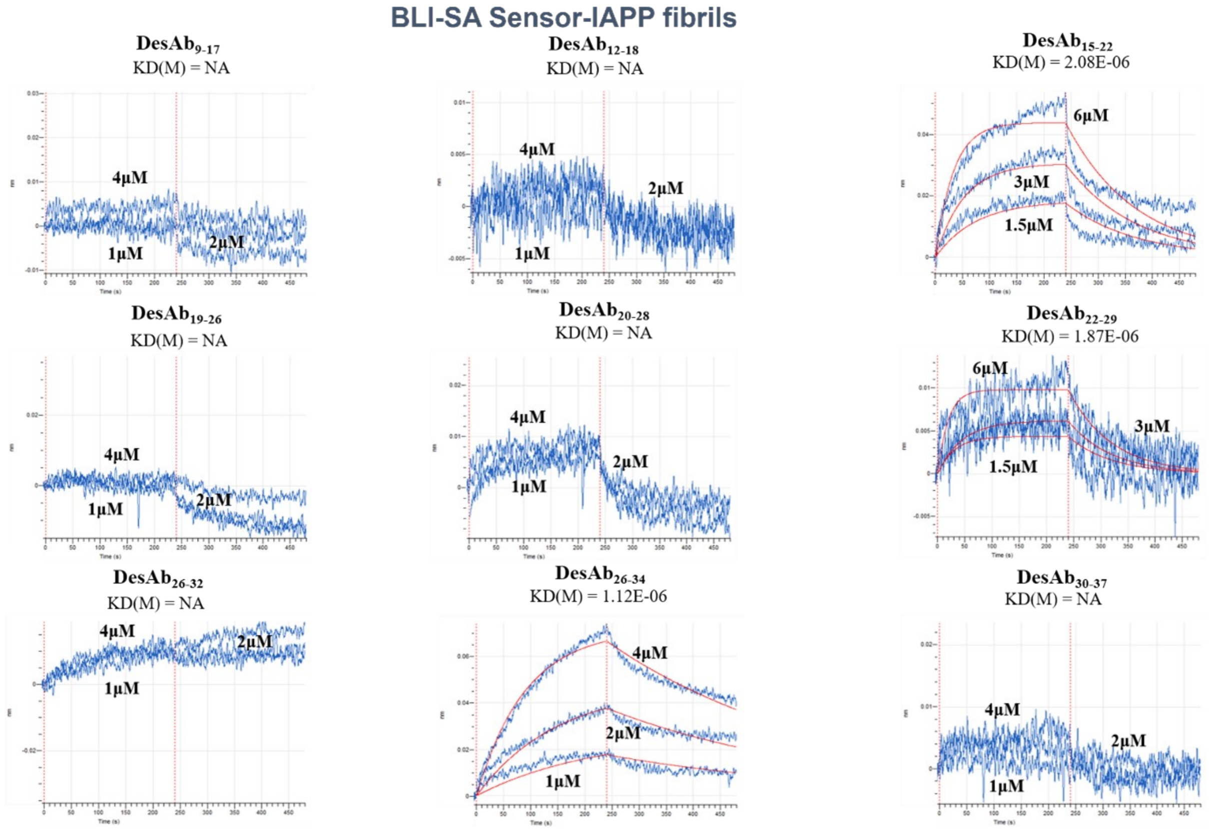

Figure S8. Measurement of the binding affinity of the DesAbs to IAPP fibrils. BLI binding traces (association and dissociation) obtained with AR2G sensors loaded with pre-prepared IAPP fibrils. Association was monitored in wells containing different concentrations of DesAbs, as shown in the figure.

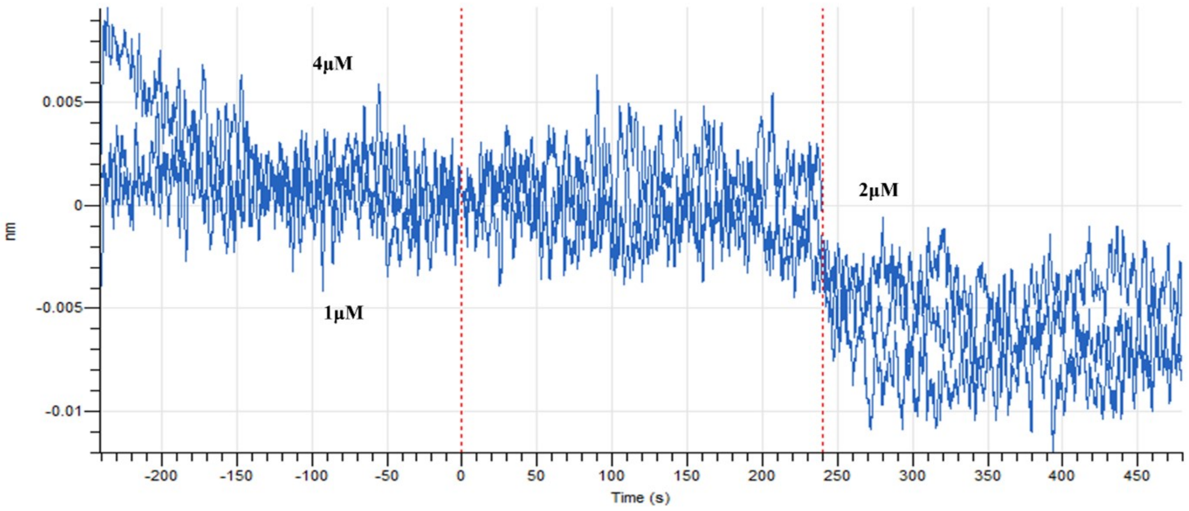

Figure S9. Measurement of the binding affinity of the negative control DesAb$_{neg}$ to IAPP fibrils. BLI binding traces (association and dissociation) obtained with AR2G sensors loaded with pre-prepared IAPP fibrils. Association was monitored in wells containing different concentrations of DesAb$_{neg}$, as shown in the figure.

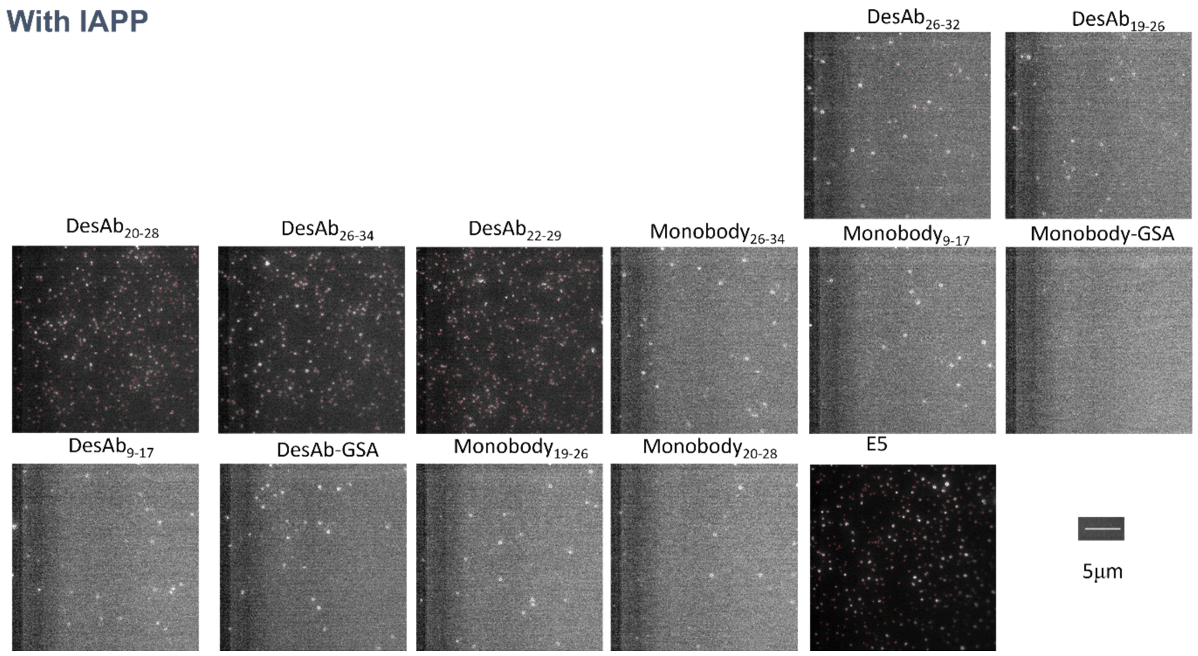

Figure S10. Images obtained for fluorophore-labelled designed antibody-like scaffolds for immobilised monomeric IAPP. Each image represents a single field of view among a total of 16 different fields of view per sample. Scale bar = 5 μm.

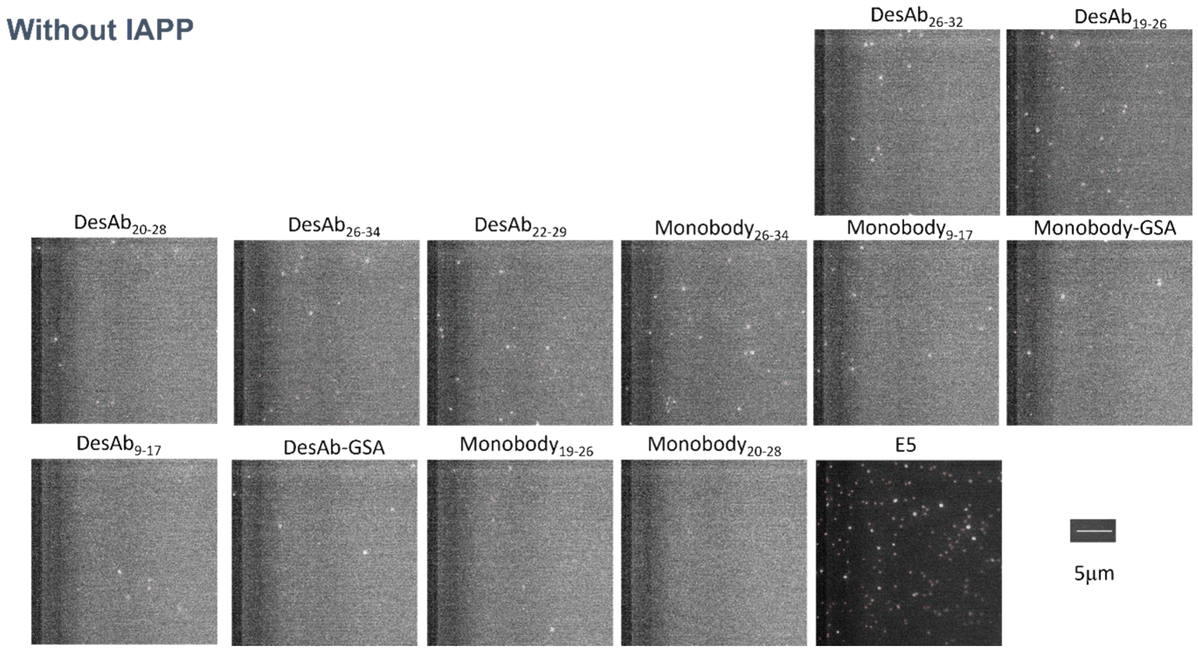

Figure S11. Images obtained for fluorophore-labelled designed antibody-like scaffolds for a buffer control (PBS, without IAPP monomers). Each image represents a single field of view among a total of 16 different fields of view per sample. Scale bar, 5 µm.

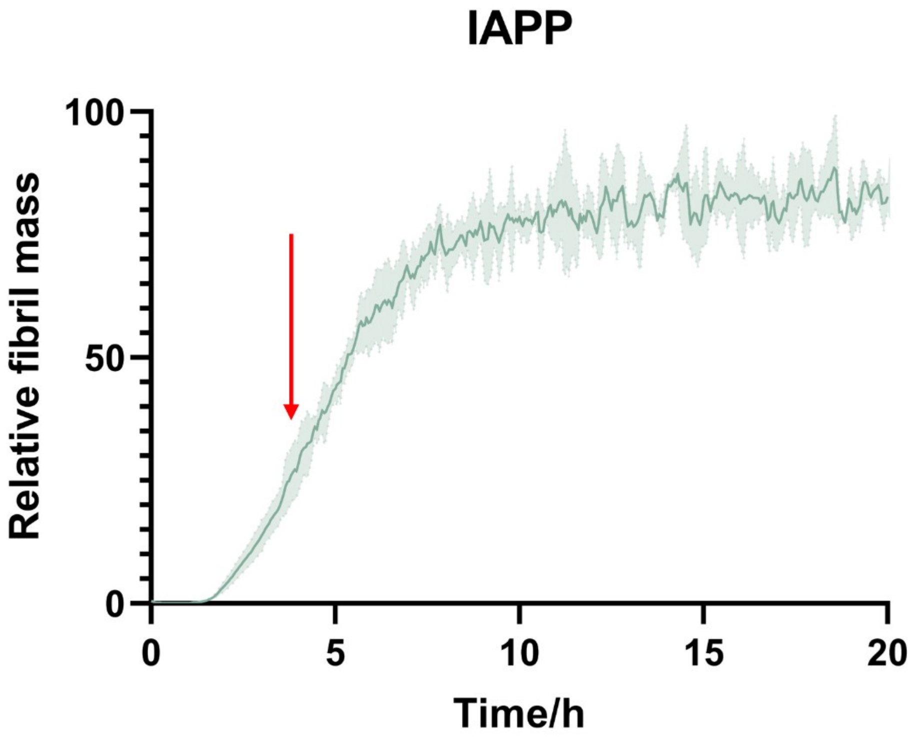

Figure S12. Aggregation assays of the IAPP (5 μM) in PBS buffer. The red arrow indicates the time point when we took out the sample and used it for IAPP aggregates imaging.

IAPP aggregates

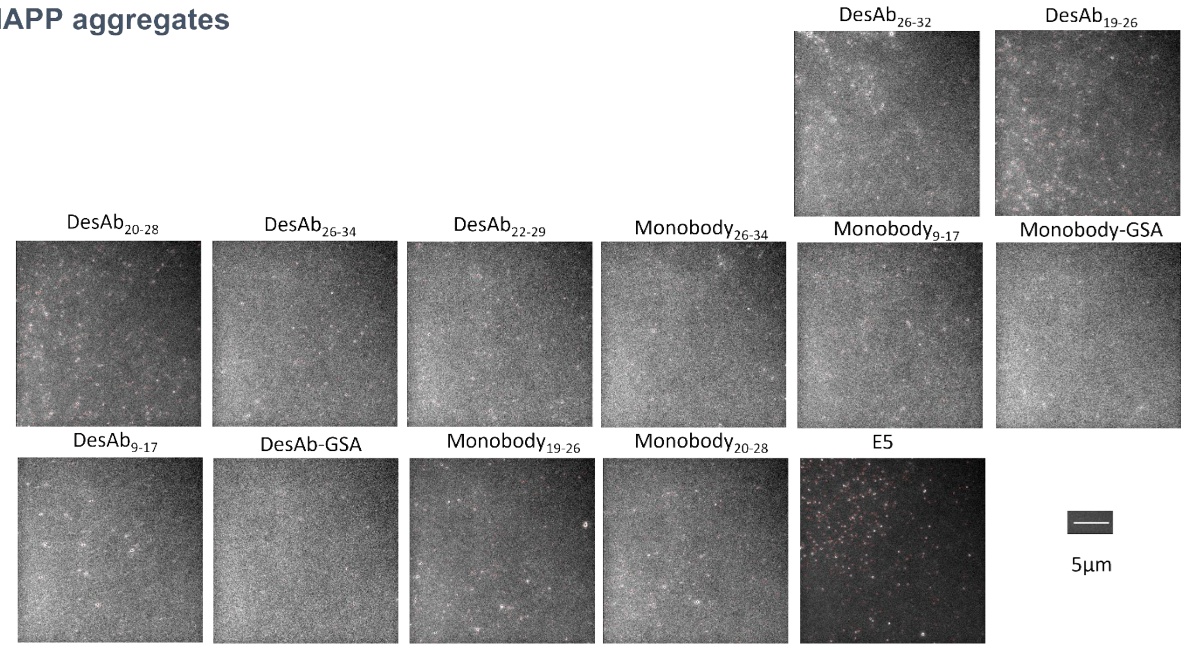

Figure S13. Images obtained for fluorophore-labelled designed antibody-like scaffolds for IAPP aggregates. Each image represents a single field of view among a total of 16 different fields of view per sample. Scale bar = 5 µm.

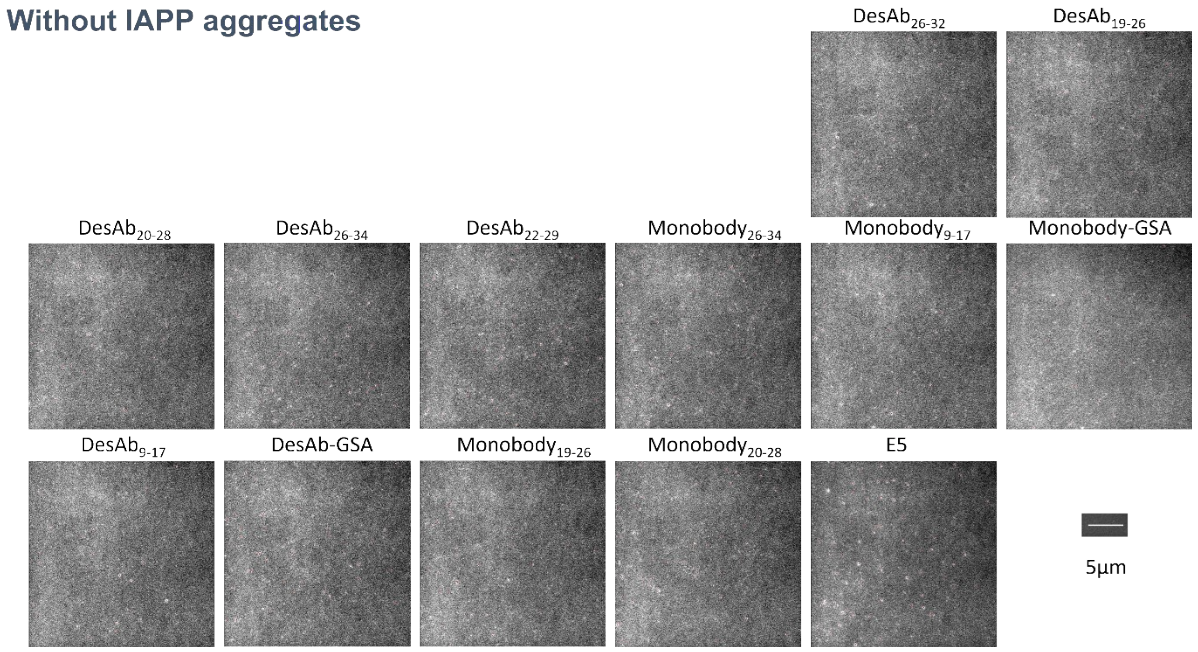

Figure S14. Images obtained for fluorophore-labelled designed antibody-like scaffolds for a buffer control (PBS, without IAPP aggregates). Each image represents a single field of view among a total of 16 different fields of view per sample. Scale bar = 5 µm.

With T2D serum

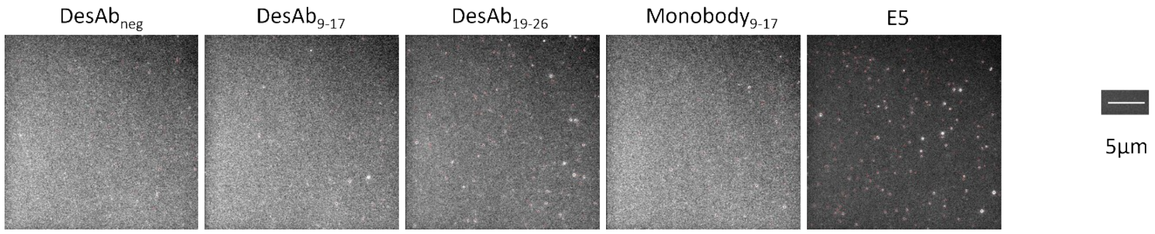

Figure S15. Images obtained for fluorophore-labelled designed antibody-like scaffolds for T2D serum. Each image represents a single field of view among a total of 16 different fields of view per sample. Scale bar = 5 µm.

Without T2D serum

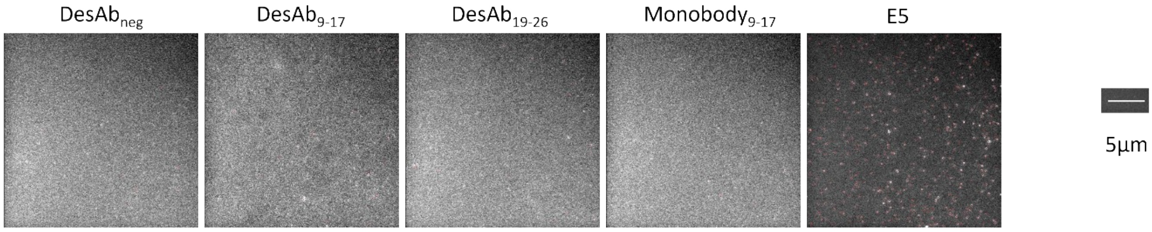

Figure S16. Images obtained for fluorophore-labelled designed antibody-like scaffolds for a buffer control (with 10% goat serum). Each image represents a single field of view among a total of 16 different fields of view per sample. Scale bar = 5 µm.

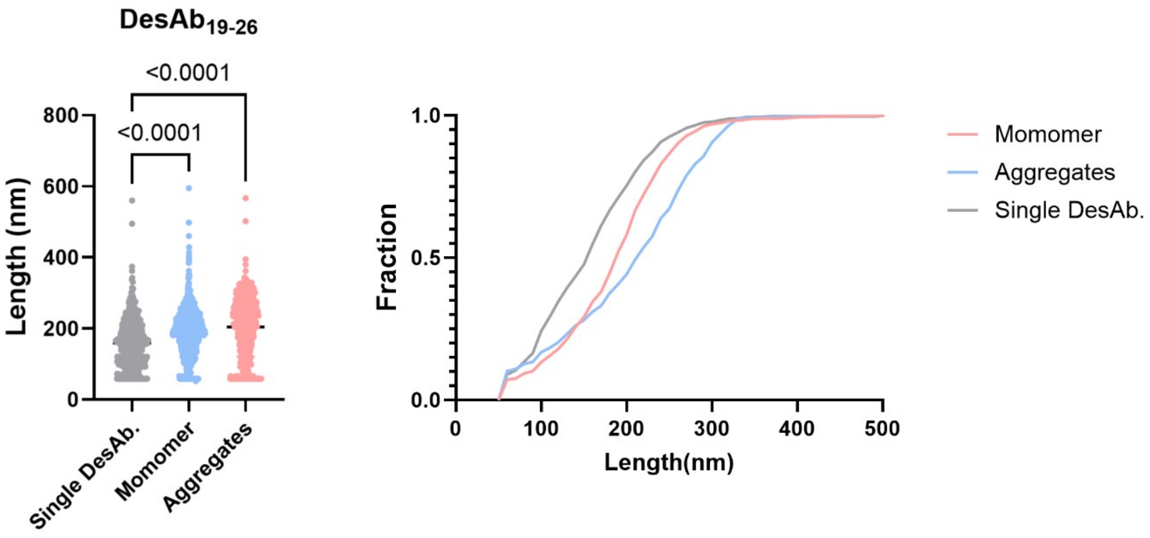

Figure S17. Length distribution of monomeric IAPP, aggregated IAPP and single DesAb. (DesAb$_{19\text{-}26}$). The size of single DesAb. Is significant smaller than monomeric and aggregated IAPP ($P<0.0001$, one-way ANOVA), indicating the SiMPull detected IAPP aggregates. When use 90th percentile of single DesAb. as the threshold, 17% of the monomeric species and 34% of the aggregated species is above the limit.

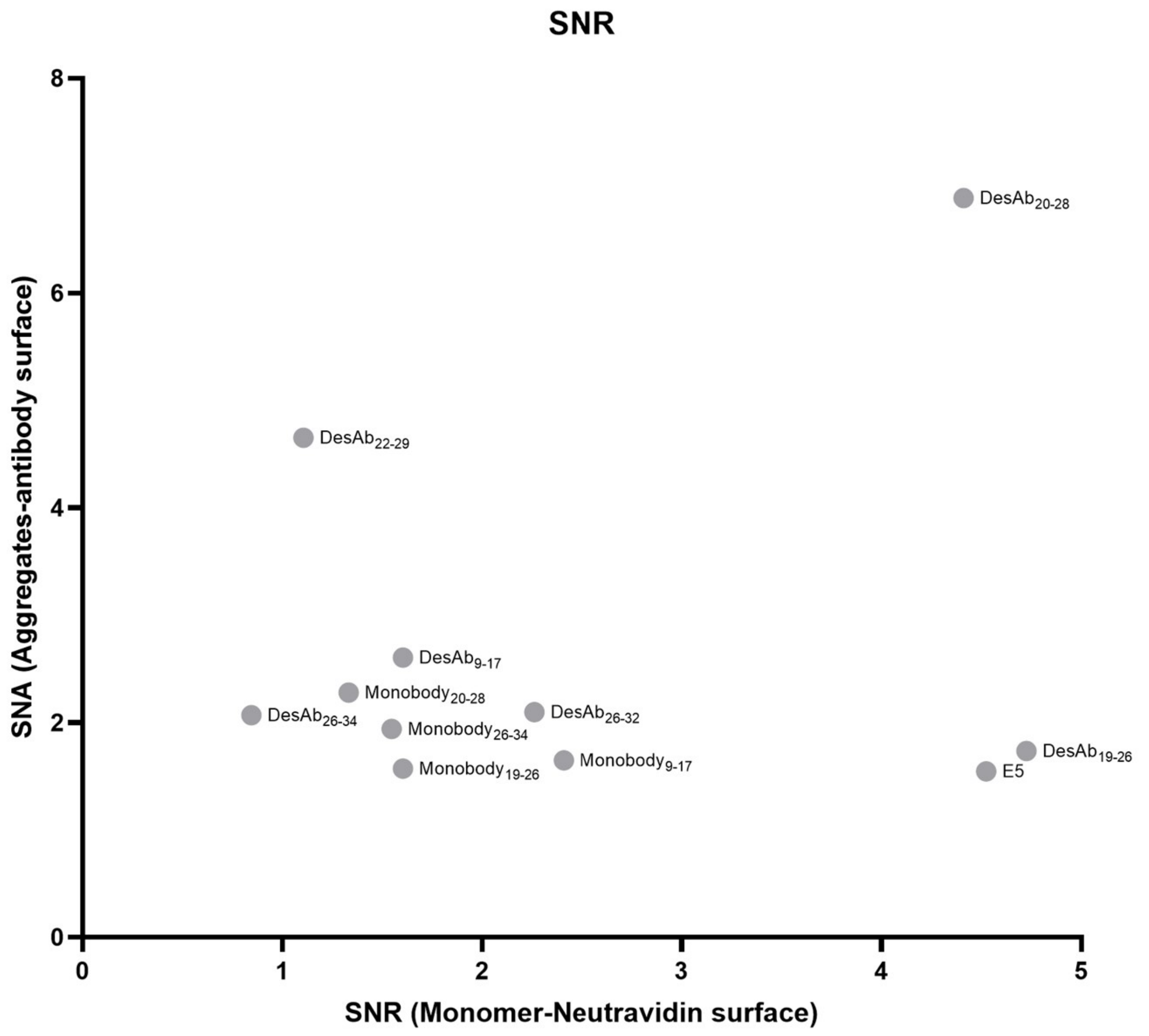

Figure S18. Signal-to-noise ratio of DesAbs. The signal-to-noise ratio (SNR) was calculated as the ratio of single-molecule counts obtained from positive samples to those from negative controls, as shown in Fig. 6. Since the capture surface for aggregates and monomers is different (see methods), the level of non-specific binding is not identical.

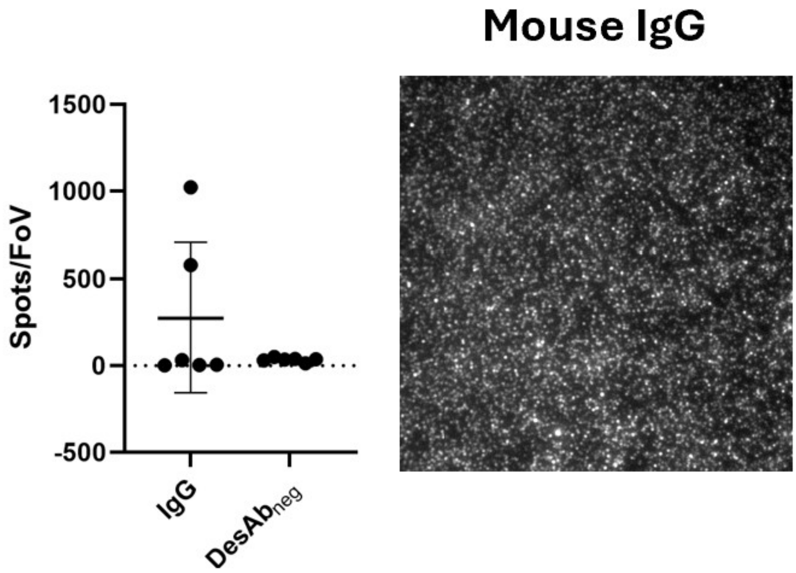

Figure S19. Non-specific binding of IgG antibodies and DesAbs. Serum samples from Fig. 8 were further tested using an IgG isotype control and a DesAbs$_{neg}$ antibody to assess non-specific interactions. Approximately 30% of samples (2 out of 6) showed strong reactivity with the IgG control, whereas no such reactivity was observed with DesAbs. The representative image for DesAbs$_{neg}$ is in Fig. S16.

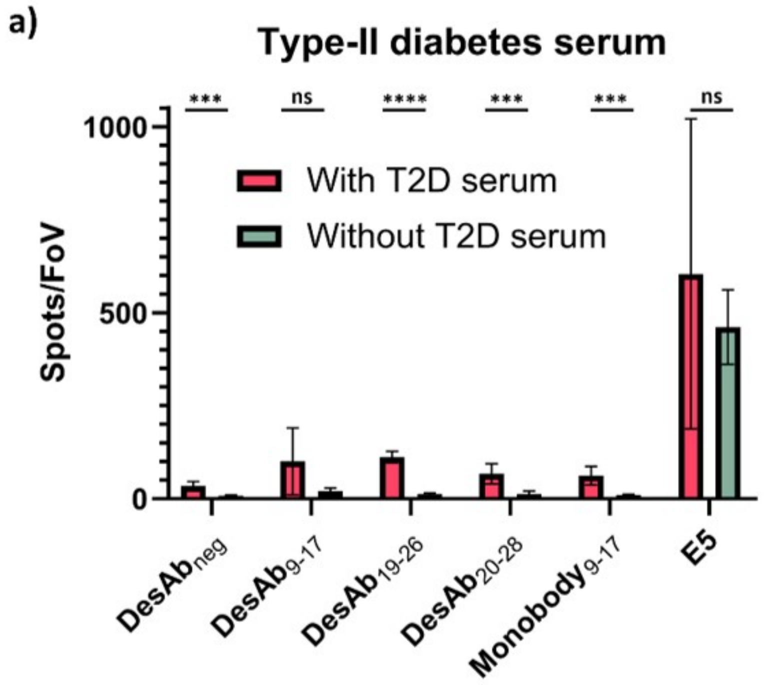

Figure S20. Single molecule IAPP detection in serum with negative controls. Detected spots per field of view for IAPP species in serum samples under TIRF microscopy by using E5 as capture antibody and different fluorescence-labelled designed antibody-like scaffolds as detection antibodies. The negative control is the signal acquired from the sample diluent (PBST).
